# Decoding Protein Stabilization: Impact on Aggregation, Solubility, and Unfolding Mechanisms

**DOI:** 10.1021/acs.jcim.5c00611

**Published:** 2025-08-06

**Authors:** Martin Havlásek, Sérgio M. Marques, Veronika Szotkowská, Antonín Kunka, Petra Babková, Jiří Damborský, Zbyněk Prokop, David Bednář

**Affiliations:** † Loschmidt Laboratories, Department of Experimental Biology and RECETOX, Faculty of Science, 37748 Masaryk University, Kotlarska 2, Brno 611 37, Czech Republic; ‡ International Clinical Research Centre, St. Anne’s University Hospital, Pekarska 53, Brno 656 91, Czech Republic

## Abstract

Modern computational tools can predict the mutational effects on protein stability, sometimes at the expense of activity or solubility. Here, we investigate two homologous computationally stabilized haloalkane dehalogenases: (i) the soluble thermostable DhaA115 (*T*_m_^app^ = 74 °C) and (ii) the poorly soluble and aggregating thermostable LinB116 (*T*_m_^app^ = 65 °C), together with their respective wild-type variants. The intriguing difference in the solubility of these highly homologous proteins has remained unexplained for three decades. We combined experimental and in-silico techniques and examined the effects of stabilization on solubility and aggregation propensity. A detailed analysis of the unfolding mechanisms in the context of aggregation explained the negative consequences of stabilization observed in LinB116. With the aid of molecular dynamics simulations, we identified regions exposed during the unfolding of LinB116 that were later found to exhibit aggregation propensity. Our analysis identified cryptic aggregation-prone regions and increased surface hydrophobicity as key factors contributing to the reduced solubility of LinB116. This study reveals novel molecular mechanisms of unfolding for hyperstabilized dehalogenases and highlights the importance of contextual information in protein engineering to avoid the negative effects of stabilizing mutations on protein solubility.

## Graphical abstract

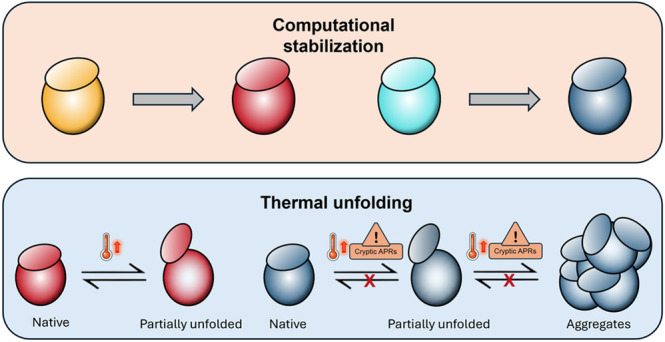

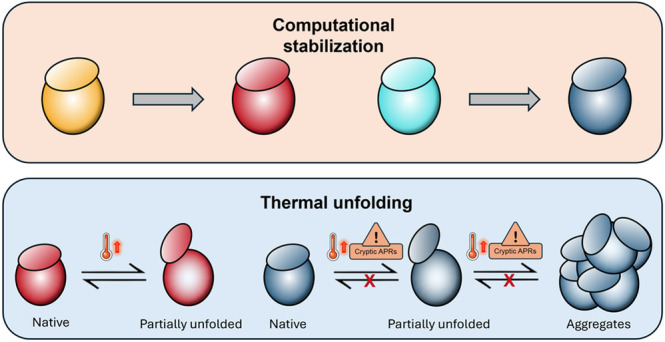

## Introduction

Stability and solubility represent two major prerequisites for the application of proteins in industry. Yet, natural proteins typically lack the stability necessary to withstand harsh conditions often required in biotechnological applications^1^ or have relatively low solubility for large-scale production and usage.^2^ Poor solubility, which is commonly associated with increased aggregation propensity, further limits the quality and performance of biomolecules in fields such as pharmacology by increasing their immunogenicity^3^ or reducing the shelf life of those proteins.^4^ The aggregation typically occurs from the partially or fully unfolded states,^5,6^ hence, increasing protein conformational stability often reduces the aggregation and improves the solubility or expression levels.^7^ That is particularly true for kinetic stabilization, which is characterized by an increase in the unfolding energy barrier that results in slowing down the irreversible deactivation process.^8^ However, aggregation may occur independently of protein unfolding.^5,6^ These aggregation pathways involve self-association of natively folded proteins, which is primarily governed by protein colloidal stability.^6^ In such cases, increased conformational stability may not suppress aggregation.^5^ Several studies have shown that stabilization may decrease the protein solubility,^9–12^ underscoring the interplay between conformational and colloidal stability in protein aggregation.

Numerous computational tools have been developed to aid in engineering recombinant proteins toward enhanced stability^13,14^ and solubility.^14,15^ The main strategies for protein stabilization include evolution-based methods, force-field calculations, machine learning techniques, or their various combinations with the single goal of finding substitutions that optimize the molecular interactions in the protein structure and stabilize its native folded form.^1^ Despite the numerous successfully stabilized proteins already reported,^16–18^ such a task remains challenging and laborious and often requires detailed knowledge of the engineered proteins. Approaches for improving solubility or reducing aggregation are typically based on the identification of aggregation-prone regions (APRs), enabling their targeted engineering to suppress the aggregation.^19^ These tools commonly employ machine learning algorithms trained to recognize characteristic features of APRs.^19^ Protein unfolding can be studied through various methods, including experimental techniques and computational simulations, each offering valuable insights into the process.^20–22^ A deeper knowledge of the protein unfolding pathway, complemented with aggregation propensity analysis may be useful for diverse purposes, including the design of enhanced variants by rational protein engineering.^23,24^ Disclosing the unfolding intermediate species and comparing them for stable and unstable variants may pave the way to further increase the stability of target proteins.^25^ On the other hand, unveiling the unfolding mechanism may help explaining different aggregation propensities^26,27^ and the expressibility of those proteins, or even allow an understanding of the reversibility of the unfolding processes.^28,29^

Haloalkane dehalogenases (HLDs, EC 3.8.1.5), enzymes facilitating the hydrolytic cleavage of carbon–halogen bonds, are excellent models for protein engineering studies for several reasons. HLDs belong to α/β hydrolase superfamily and are highly relevant for biotechnology applications.^30^ HLDs were previously engineered for activity,^31^ specificity,^31^ enantioselectivity,^32^ and stability.^33–35^ The globular structure of HLDs is composed of two domains—an evolutionary conserved main domain and a more variable cap domain. While the cap domain is formed entirely of α-helices, the main domain consists of a β-strand core surrounded by α-helices.^36^ The helical and flexible cap domain plays an essential role in the substrate specificity, as it strongly affects the shape of the molecular tunnels, ensuring the transport of the reactants and products into and out of the protein active site.^37^

Here, we describe the effects of the stabilization of two well-studied HLDs on their solubility and aggregation propensity. These HLDs share a high sequence identity (∼50%), structure similarity (root-mean-square deviation, RMSD = 0.63 Å for Cα atoms), and similar thermal stability. However, their soluble expression levels and aggregation propensities differ significantly. These differences were further exacerbated upon their stabilization. This study aimed to investigate the mechanisms of protein unfolding and aggregation in order to explain such observed differences in their solubility and aggregation propensity.

## Results and Discussion

### Overview of Wild-type and Hyperstable Enzymes

DhaAwt (UniProt ID: P0A3G3), a well-soluble dehalogenase purified in high yields (85 mg/L of cell culture), was successfully stabilized using the FireProt workflow, which resulted in the highly stable multiple-point mutant DhaA115 ^35^ (*T*_m_^app^ = 74 °C). The combination of 11 introduced mutations (Figure 1A, Table 1) enhanced the thermostability by 23 °C, making it the most stable HLD known at the time, without significantly compromising its solubility or aggregation propensity (Figure 1B, Tables 1 and S1). The following comprehensive investigation of its crystal structure revealed the stabilizing effect of the mutations resulting from the reduction of tunnel volumes and changes in the protein backbone that optimized the compactness of the structure.^38^ Moreover, the newly designed DhaA115 allowed for further stabilization rounds that yielded extremely stable dehalogenases with increased catalytic properties.^33^ In contrast, LinBwt (UniProt ID: D4Z2G1), an enzyme attractive for its capability to degrade cyclic substrates,^39^ tends to aggregate, and its soluble expression is relatively low (15 mg/L of cell culture). Its stabilization yielded highly stable LinB116 (*T*_m_^app^ = 65 °C) but at the cost of decreasing its soluble expression level and increasing the aggregation propensity (Figure 1B, Tables 1, and S1). LinB116, reported here for the first time, combines 8 stabilizing mutations designed previously by FRESCO^34^ and an extra stabilizing mutation L177W, which partially closed one of the access tunnels and modulated the substrate specificity^40^ (Figure 1A, Table 1).

**Figure 1:**
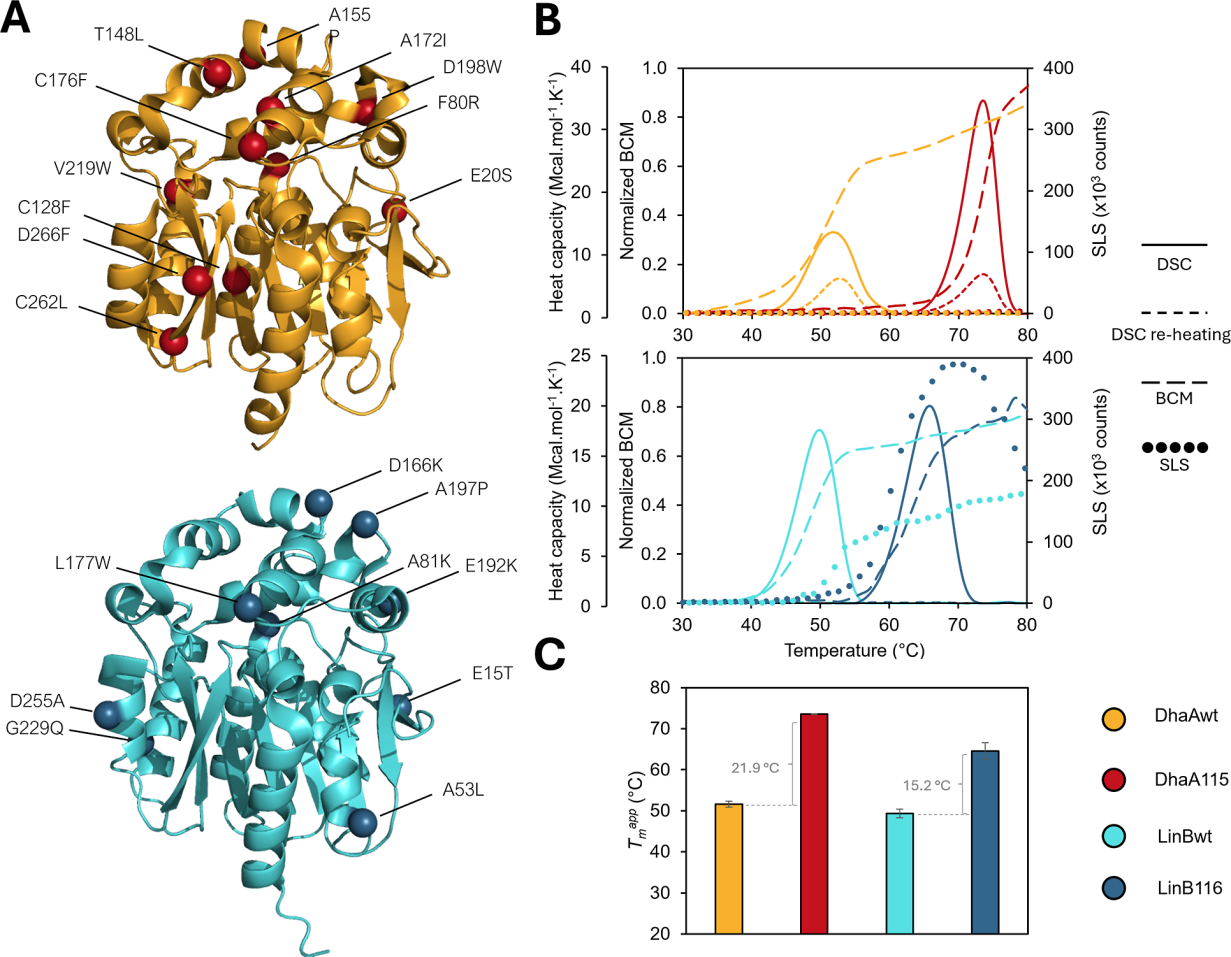
Structural and biophysical characterization of DhaAwt, LinBwt, and their stabilized mutants. (A) Structure overview of DhaAwt (gold) and LinBwt (light blue) with the respective stabilizing mutations introduced into DhaA115 (dark red spheres) based on FireProt^35^ and LinB116 (dark blue spheres) based on FRESCO.^34^ (B) The overview of the thermostability experiments. The calorimetric thermograms (full line) display the heat capacity of the unfolding, and reheated scans (short-dashed line) show the unfolding reversibility of the examined proteins. The barycentric mean (BCM) curves (long-dashed line) correspond to the gradual protein unfolding, and the static light scattering (SLS) data (dotted line) display the aggregation. (C) The bar graph shows the experimentally determined melting temperatures. The values were calculated as the average of the values obtained using DSC and DSF. The error bars represent the standard deviation.

**Table 1:** Overview of Properties of the Tested HLDs^a^

variant	mutations	*T*_m_^app^ (°C)	Δ*T*_m_^app^ (°C)	Ag*g*_max_ (counts)	Unfolding rev. (%)
DhaAwt	NA	51.6 ± 0.7	NA	1925 ± 408	29%
DhaA115	E20S + F80R + C128F + T148L + A155P + A172I + C176F + D198W + V219W + C262L + D266F	73.6 ± 0.1	21.9 ± 0.8	4278 ± 298	20%
LinBwt	NA	49.3 ± 1.1	NA	181294 ± 477	0%
LinB116	E15T + A53L + A81K + D166 K + L177W + E192 K + A197P + G229Q + D255A	64.6 ± 2.0	15.2 ± 3.1	386349 ± 4397	0%

a Stabilizing mutations, melting temperatures, aggregation, and unfolding properties. The apparent melting temperature (*T*_m_^app^) was calculated as the average of the respective measurements by DSC and DSF, the maximal aggregation (Agg_max_) describes the top of the static light scattering curve between 20 and 80 °C, and the unfolding reversibility (Unfolding rev. in %) describes the fraction of the refolded protein after heating the sample to the temperature right after the endothermic calorimetric peak in the first run. NA–not applicable. The uncertainty values represent the standard deviation.

#### Biophysical Experiments Revealed Differences in Aggregation and Unfolding Reversibility

Thermal stability of both wild-type dehalogenases (DhaAwt and LinBwt) was previously determined to reach similar levels in terms of their apparent melting temperatures and was successfully pushed forward using computational stability engineering^34,35^ (Figure 1B,C). The melting scans of all protein variants measured by differential scanning fluorimetry (nanoDSF) were found to be independent of protein concentration in the range of 0.1 to 1.0 mg/mL (Figure S2, Table S2). Reheating scans using differential scanning calorimetry (DSC) revealed the complete irreversibility of the unfolding of both LinB variants, while the two DhaA variants maintained some levels of reversibility after the complete unfolding (Figure 1B, Table 1). However, the reversibility of DhaA115 was slightly reduced compared to the wild-type (Figure 1B, Table 1). The unfolding signal of the LinB variants was accompanied by a large increase in scattering intensity, indicative of aggregation, more prominent in the stabilized LinB116 (Figure 1B). Notably, the aggregation profiles differed between LinBwt and its stabilized variant. In LinBwt, the aggregation onset followed the unfolding signal, whereas the opposite trend was observed for LinB116. These observations highlight the persistence of aggregation despite successful conformational stabilization and suggest that LinB116 aggregation is likely driven by low colloidal stability (based on the *T*_onset_ of aggregation) rather than conformational instability (*T*_m_^app^). The stabilizing effect of the introduced mutations was accompanied by increased surface hydrophobicity of LinB116, contributing to its poor yield and solubility (Tables S1 and S5). To further elucidate the underlying aggregation mechanisms, we performed global analysis of unfolded experiments described in the next section.

### Global Analysis Highlights the Differences in the Unfolding Mechanisms

The thermal unfolding data were analyzed globally to reveal quantitative and mechanistic insights on the unfolding processes of DhaAwt, LinBwt, and their stabilized variants (Figure 2) using the CalFitter 2.0 Web server.^41^ Guided by previous studies,^33^ an unfolding model involving one intermediate state was selected as the most biologically relevant for all four HLDs (Figure 2C). Despite being partially reversible, the best overall fit of DhaAwt and DhaA115 data was obtained using a three-state irreversible model due to the limited experimental data on the reverse (folding) rate. The unfolding of all four proteins shows strong scan-rate dependency (Figure 2A), which, together with the generally poor reversibility, suggests that the unfolding is under kinetic control.^8^ The model is parametrized in terms of calorimetric enthalpy (Δ*H*_cal_) and the change in the Gibbs activation energy of unfolding (Δ*G* ^⧧^(*T*)). Interestingly, despite having similar *T*_m_^app^, the calorimetric enthalpy of LinBwt is higher than that of DhaAwt, indicating a potential entropic contribution to the stability in the case of DhaAwt (Figure 2F).

**Figure 2:**
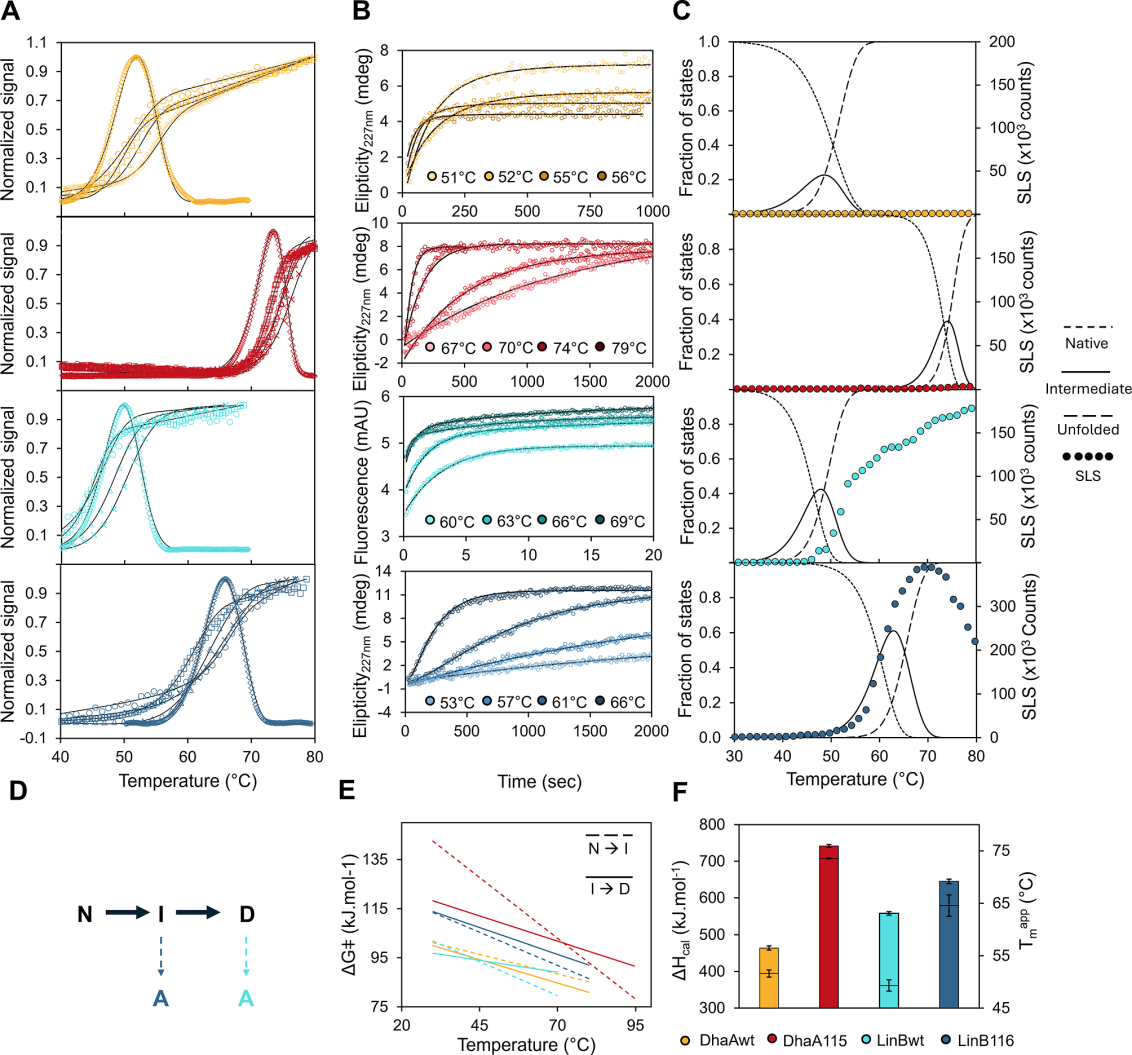
Global fitting of the unfolding data for DhaAwt, LinBwt, and their stabilized variants. (A) The global fitting of the temperature scanning data. The plot shows the experimental data from DSC (diamonds), nanoDSF data in 3 scan rate settings (0.3/0.5 °C/min–squares, 1 °C/min–triangles, and 2 °C/min–crosses), and CD (circles). The respective modeled data are shown as black lines. (B) The global fitting of the unfolding kinetics was measured in the range of 50–80 °C. The plot shows the experimental data (circles) and the respective modeled data (black lines). (C) The superimposition of the SLS signal (circles) with the modeled fraction of states highlights the main aggregation pathways of LinB variants. (D) The irreversible 3-state unfolding model was selected for the global fitting of the experimental data (native–N, intermediate–I, and denatured–D). The supposed main species driving the aggregation (A) of LinBwt and LinB116 are highlighted by the light and dark blue dashed arrows, respectively. (E) The comparison of the energy barriers of the first and second steps of the unfolding shows different behavior between DhaA and LinB variants. (F) The comparison of the calorimetric enthalpy (Δ*H*_cal_, colored bars) and the melting temperature (*T*_m_^app^, black lines in the corresponding bars) indicates the entropic contribution to the stability of DhaAwt. The error bars represent the standard deviation.

The analysis revealed differences between the barriers of the first (Native → Intermediate) and second (Intermediate → Denatured) unfolding steps in the DhaA and LinB variants (Figure 2E). While in the case of DhaAwt and its stable variant, the first energy barrier is higher than the second one in most of the temperature range, and thus limiting the unfolding rate, in the case of LinBwt and mainly LinB116 it is the opposite. That might potentially lead to a higher accumulation of the (partially) unfolded intermediates that may be prone to aggregation. These results are consistent with the previous experimental findings, where the aggregation of LinB116 preceded unfolding to a greater extent than observed for LinBwt. This hypothesis is further supported for LinB116 by overlaying the SLS signal with the modeled fraction of states derived from global fitting analysis (Figure 2D). In the case of LinB116, the rising of the light scattering signal strongly correlates with the accumulation of the intermediate species, which might indicate that the accumulation of the unfolding intermediate is one of the main drivers of its aggregation. In the case of LinBwt, the SLS signal follows the accumulation of the unfolded species, suggesting that LinBwt starts to aggregate mainly after the unfolding. These findings indicate that the mutations present in LinB116 have altered the primary aggregation pathway, causing it to initiate from an unfolding intermediate rather than from a fully unfolded state, as predominantly observed with LinBwt.

### In Silico Protein Unfolding Identifies Cryptic Aggregation-Prone Regions

We used molecular dynamics (MD) simulations to complement the experimental results and gain structural insights into the thermal unfolding of the four HLDs. We applied adaptive sampling to survey the dynamical conformational space of those proteins. This method consists of running several MDs in parallel, over multiple consecutive batches, or epochs, in an adaptive manner to progressively explore more diverse conformations of the protein along the simulation time. The MDs from each epoch are iteratively seeded from selected snapshots from previous MDs based on a specified criterion. In this case, the RMSD of the C_α_ atoms was the feature, or metric, used to build on-the-fly Markov state models (MSMs) and guide adaptively the MD simulations.^42,43^ In a combined simulation time of ca. 20 μs, we surveyed a considerable range of RMSD values in all four protein systems (Figure S3). The simulations were carried out at 98 °C, which is above the melting temperature to accelerate the computational sampling of the unfolding process. According to previous evidence, this approach does not alter the unfolding pathway.^44,45^

MSMs were constructed based on the same RMSD metric to analyze the conformational diversity sampled in the MDs, resulting in satisfactory quality models with three and four states for the DhaA and LinB variants, respectively (Figures S4 and S5). The respective clusters (or macrostates) were used to define three main states: folded, intermediate, and unfolded (Figures 3 and S6, Table S3). This number of conformational states was selected to match the kinetic model described above. The folded (or native) state corresponds to the cluster with the lowest mean RMSD value, the unfolded state (or partially unfolded) has the highest RMSD, and the intermediate state is in between. The unfolded states contain the most extreme conformations (especially for DhaAwt), but because they are most likely only partially unfolded and the simulated systems are not in equilibrium (i.e., at 98 °C we expect that simulating longer times would increase the unfolded populations), we considered them only for qualitative purposes. We want to clarify that the intermediate and unfolded populations obtained from our MDs do not necessarily correspond to the experimental states described in the previous section. In our simulations, we sampled only the early stages of the protein denaturation, and we expect that more extensively unfolded conformations will occur at later stages of the unfolding process. Nevertheless, we assume that this intermediate is a transient state between the folded and more extensively unfolded conformations. As such, the intermediate state can determine the development of the unfolding process, and hence it can be valuable to explain some of the differences between the various proteins, and to design improved variants. Noteworthy is the fact that DhaAwt and DhaA115 are experimentally known to preserve a significant amount of secondary structure after unfolding, which is in agreement with the results from our simulations (Table S4). This cannot be verified for the LinB variants due to their aggregation. Analyzing in more detail the different states, we find that the populations of the folded states were rather low compared to the remaining states (see Table S3 and Figure S7A). This is not surprising, considering the high temperature employed in the MDs. Nonetheless, the folded states were more populated for the stable variants than for the respective wild-types, which is in agreement with the experimental ranking in terms of stability (Table 1). Interestingly, the mean RMSD in each state was always similar or lower for the stable variants compared to their wild-types (Table S3 and Figure S7B). This means that, on average, the more stable variants tend to drift less extensively from the native state than their less stable wild-types.

**Figure 3:**
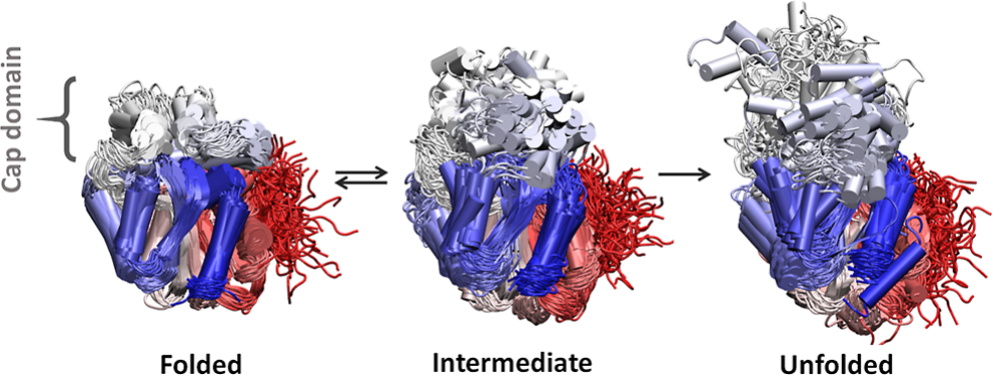
Conformational states observed during the unfolding process. Clusters obtained from the MSMs analysis of the MD simulations of DhaA115 describing the folded, intermediate, and unfolded states. The protein is represented in the cartoon, the red-white-blue color gradient shows the residue sequence index (red = N-terminus, blue = C-terminus), and the cap domain (ca. positions 135–215, white region) is labeled. Each state is represented by 100 randomly selected frames superimposed and aligned to the crystal structures.

For all studied proteins, the regions that changed the most during the unfolding process were the cap domain (positions 135–215), loop L5–helix α2 (positions 65–80), loop L16–helix α9 (positions 240–260), and strand β8–loop L18–helix α10 (positions 265–280). For simplicity, all the secondary structure elements mentioned here refer to the DhaAwt conventional labeling. The unfolding regions were assessed by (i) comparing the structures of different state ensembles (Figures 3 and S6), (ii) calculating the global RMSD by residue (Figure S8), and (iii) analyzing the most flexible regions in the folded and intermediate states (Figures 4, S9 and S10). Unfolding of the cap domain affected loop L5 since they are in close contact. A similar effect occurred with loops L16 and L18 (Figures 4 and S10). These results agree with previous studies showing that the cap domain of DhaA115 is prone to thermal unfolding.^33^ Those same regions also showed high fluctuation within each state, particularly in the intermediate and unfolded states, demonstrating their strong destabilization during the unfolding (Figures 4, S9 and S10). Loop L5 was the region that differed the most between the different enzymes, as it was remarkably more stable in the LinB variants (especially in LinB116) than in the DhaAs. Interestingly, in the folded state, the stable DhaA115 was slightly more flexible than DhaAwt in the regions that unfolded first (especially in the cap domain), and the opposite was observed for the intermediate states (Figure 4). This suggests that an increased entropy of the native state of DhaA115 contributes to its enhanced conformational stability, which is in line with what has been observed experimentally.^46^ In contrast, LinB116 is less flexible than LinBwt, suggesting a stabilization via rigidification of the native state. There have been discussions on the importance of flexibility and rigidity to the stabilization of a protein, and the delicate balance between those opposite effects makes it challenging to rationalize and predict the dominant outcome.^47–49^

**Figure 4:**
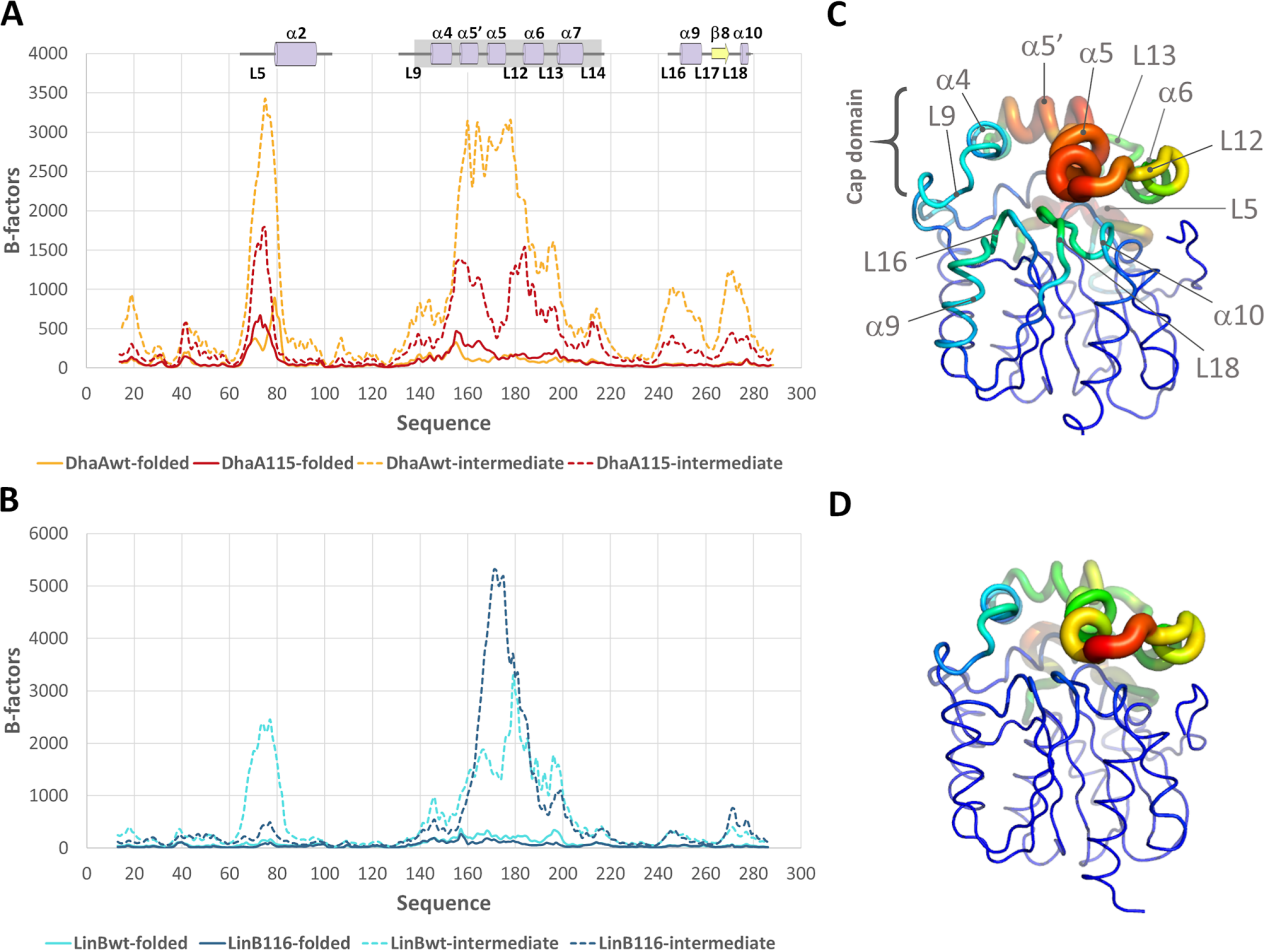
Conformational flexibility driven by the unfolding process. (A) and (B) *B*-factors of the backbone atoms (C, C_α_, N, O) of each residue within the folded and intermediate states, superimposed for the DhaA (A), and the LinB (B) variants. The regions of the sequence involved in the unfolding are labeled for DhaA variants on top of the respective graph. (C,D) Putty representation of the *B*-factors in the intermediate states of DhaAwt (C) and LinBwt (D). The hottest colors (red) and thickest tubes correspond to the highest *B*-factor values, and the coldest (blue) and thinnest to the lowest values; the main flexible regions highlighted in (A) are labeled for DhaAwt. The structures displayed correspond to the crystal structures of DhaAwt (PDB ID 4E46) and LinBwt (PDB ID 1MJ5).

In the next stage, we calculated the absolute and relative solvent-accessible surface areas (SASA and rASA, respectively) for all residues in all the different states of all four proteins (Table S3 and Figure S7C). We found that (i) expectedly, SASA and rASA increased from the native to the non-native states as a result of a higher exposure of natively buried residues to the solvent during unfolding; and (ii) in the intermediate states, the stabilized variants showed lower SASA and number of exposed residues than the less stable wild-types, revealing a higher compactness of the former along the unfolding pathway. We also analyzed which residues changed their solvent exposure status (i.e., crossed the threshold of rASA = 0.25) when going from the native to the intermediate state. This allowed for identifying the regions that became more exposed or buried in that process (Figures 5, S7D and S11). The regions that became more exposed include the most flexible ones mentioned above (cap domain, loop L5, helix α9), but also some others that were in contact with them (e.g., loop L17, strand β8, loop L18 and helix α10, corresponding to positions 260–280; Figure 4). These positions are in agreement with previously reported hydrogen–deuterium exchange mass spectrometry (HDX-MS) results obtained for DhaA115.^33^

Expectedly, the more stable proteins exposed fewer residues during the unfolding than the less stable ones (Figure 5). However, the different proteins revealed considerably different patterns in the solvent-exposure changes along their sequence. The DhaA variants showed increases in solvent exposure more scattered along the sequence than the LinB variants, in which they seemed to be more localized. More remarkably, some of the regions–specifically, helix α5 (positions 160–185) and the patch of strand β8–loop L18–helix α10 (positions 260–280)—that become more exposed in the LinBs also coincide with aggregation-prone regions (APRs), according to the AggreProt^50^ predictions, which is not the case for the DhaAs (Figure 5). These differences may explain the significantly higher aggregation propensity of the LinB variants upon unfolding compared to the DhaA variants (see the section below).

**Figure 5:**
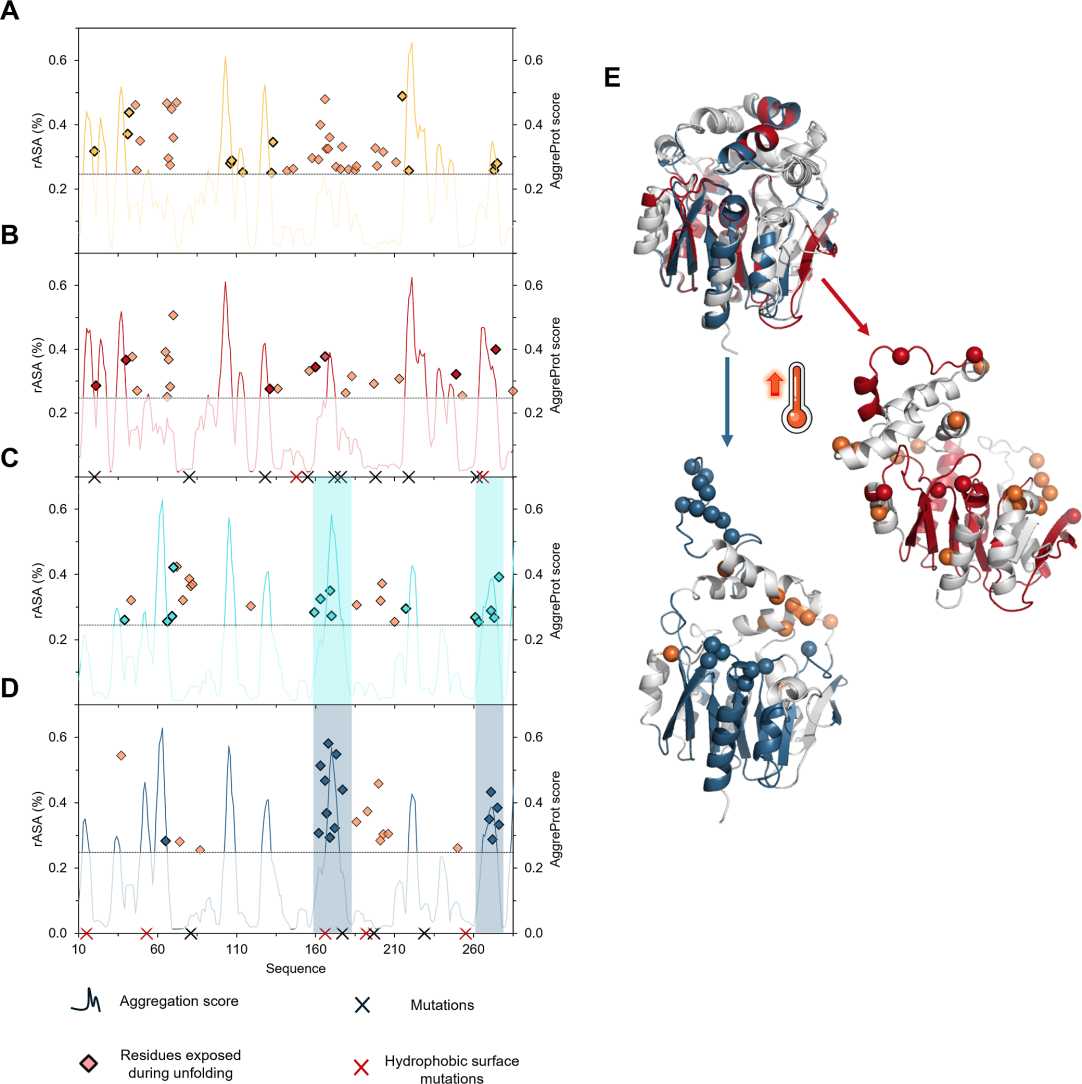
Overview of the aggregation propensity predictions and residue exposure during unfolding. (A) DhaAwt, (B) DhaA115, (C) LinBwt, and (D) LinB116. The aggregation propensity quantified by the AggreProt^50^ score is represented by the color-coded curves, and rASA of the residues that became exposed during the first step of the unfolding is represented by the diamonds. The orange diamonds stand for those residues that are not located in any predicted APR, the residues exposed during unfolding that are in some of the APRs are highlighted by the color of the respective protein (i.e., gold for DhaAwt, dark red for DhaA115, light blue for LinBwt, and dark blue for LinB116). The mutations introduced into the stabilized protein variants are marked with crosses in the sequence, where the red indicates the mutations on the surface increasing hydrophobicity. The gray dotted line represents the aggregation score threshold of 0.25 for the APRs classification; the regions that did not exceed the threshold are blurred to highlight only the APRs. The color-shaded areas highlight the regions in the LinB variants that were predicted as APRs and contained residues that became exposed during the first step of the unfolding simulations. (E) Structures showing the APRs in DhaA115 (dark red) and LinB116 (dark blue), some of which get more exposed upon thermal unfolding (representative structures are shown); the residues that became exposed during unfolding (diamond points in plots B and D) are represented as spheres: orange if they are not located in any APR, dark red if they are located in an APR in DhaA115, and dark blue if there are in an APR in LinB116.

### Mutations in Aggregation-Prone Regions Explain Stability and Solubility Trade-off

It has been previously observed that some of the stabilizing mutations on the protein surface may unintentionally lead to a decrease in protein solubility.^9–12^ This is partially because the stability predictors generally prefer hydrophobic mutations.^9,10^ Thus, we analyzed the mutations introduced to DhaAwt and LinBwt to generate the stable variants DhaA115 and LinB116 and examined their location and the changes in the hydrophobicity (indicated in Figure 5B, D by the color of the crosses in the sequence, and Table S5). This revealed the different characteristics of the mutations introduced into the two tested dehalogenases. While in the case of DhaA115, only 4 out of 11 mutations were located at the exposed positions, in the case of LinB116, 7 out of 9 were located on the surface (Figure 5B,D, Table S5). Additionally, we evaluated changes in surface hydrophobicity upon stabilization using two different hydrophobicity scales. In both cases, the stabilizing mutations led to an increase in surface hydrophobicity. While this effect was minimal in DhaA115 (0.1% and 4%, respectively), the mutations in LinB116 resulted in a more pronounced hydrophobicity increase (4% and 21%) (Figure 5B, D; Table S5). These findings highlight a substantial increase in the total surface hydrophobicity of LinB116, which may contribute to its poorer solubility, reduced expression levels, and increased aggregation propensity.

To further explore the mutational effect on the solubility and aggregation propensity of the stabilized variants, we predicted the aggregation propensity in all the variants using the newly developed aggregation predictor AggreProt^50^ (Figures 5 and S12). The mutations did not dramatically alter the overall shape of the predicted aggregation profiles in any case. However, in both cases, the stabilized variants displayed a slightly increased aggregation propensity in some regions (Figure S12), suggesting only a minor increase in the aggregation propensity of the native states of the stabilized enzyme variants. However, given the global analysis findings indicating that aggregation is predominantly initiated from the intermediate (LinB116) or unfolded state (LinBwt), we have further analyzed the predicted aggregation propensity in the context of the positions that became exposed during the first phase of the unfolding. Such analysis served to reveal potential cryptic APRs—regions that remain buried in the native state but become exposed during unfolding, allowing them to induce the aggregation (Figure 5). The results show major distinctions between DhaAwt, LinBwt, and their stabilized variants. Upon unfolding, DhaAwt exposes residues that largely do not overlap with the predicted APRs, with only 32% of exposed residues located within APRs. In contrast, LinBwt shows a higher overlap, with 56% of newly exposed residues being within predicted APRs. Moreover, these residues are more locally concentrated, primarily in two APRs (positions 160–185 and 260–290; shaded areas in Figure 5C,D), indicating the presence of potential cryptic APRs. This trend becomes even more pronounced upon stabilization of the wild-type HLDs. Upon unfolding, DhaA115 exposed fewer residues compared to its wild type, with a similar proportion (32%) located within predicted aggregation-prone regions (APRs). In contrast, 60% of the residues exposed during unfolding of LinB116 were primarily located within the two APRs described above (Figure 5).

To investigate the importance of cryptic APRs exposed upon unfolding, we have attempted to engineer these regions in LinB116 to lower its aggregation propensity. The relevance of the APRs predicted using AggreProt was further validated using the analysis of the LinB116 intermediate structures by AggreScan 3D 2.0 ^51^ (Figure S13, Table S6), which detected APRs formed by sequentially distant residues using structural input. The AggreScan 3D predictions were in strong agreement with those from AggreProt (Figure S13), as most of the aggregation hotspots identified using AggreScan 3D were localized in one of the previously predicted cryptic APR (i.e., positions 160–190). We designed and experimentally tested 11 new variants, including 6 multiple-point and 5 single point mutants of LinB116 (Table S7). All designed mutations (Table S7) destabilized the structure of LinB116 (Figure S14, Table S8), in some cases to the extent that soluble expression could not be observed (i.e., multiple-point mutations), as mostly expected given that the targeted residues are buried in the native state. The yields of the soluble designs were comparable to the LinB116 template. Notably, two single point mutants, V168G and V168 K, showed slightly reduced scattering intensity (Figure S14B). Although relatively marginal improvement, the positive effects of these mutations show promise that reducing aggregation propensity of cryptic APRs, albeit challenging, is possible. These findings may serve as a foundation for future studies aiming for greater improvements through more sophisticated strategies that integrate evolutionary insights with advanced computational and machine learning tools. The importance of these regions for the solubility of LinB structure was further demonstrated in our previous study,^52^ where the aggregation propensity and protein yields of WT were significantly improved upon mutations of exposed hydrophobic residues in these regions to hydrophilic ones. That study also showed a positive effect of mutations in other APRs (positions 217–227), which do not overlay with the residues exposed during the unfolding. That suggests that the aggregation and poor solubility of LinB variants are of a more complex nature, which arguably involves at least two independent aggregation pathways. The first is triggered by the APRs exposed in the native state that were previously identified and engineered toward higher protein solubility.^52^ The second pathway, which is described in this study, involves aggregation initiated from partially unfolded states and their cryptic APRs.^53–55^ This secondary route is likely responsible for the irreversibility of the thermal denaturation of the LinB variants, since once the protein forms the unfolding intermediate it exposes the cryptic APRs that lead to its fast aggregation. Further engineering of these cryptic regions may suppress the aggregation propensity of LinB116 upon unfolding, and potentially even improve its soluble expression, but great care must be taken not to disrupt the delicate balance of the forces stabilizing the core of the cap domain. These results highlight the complexity of the trade-off between structural stability and aggregation in some enzymes^9–12^ and point out the importance of contextual information in protein engineering projects.^56–58^

In summary, our computational approach offers a promising strategy to identify cryptic APRs that may contribute to protein aggregation upon partial unfolding. This methodology can be applied to other proteins of biotechnological or medical relevance. The main bottleneck is the MD sampling of unfolded and partially unfolded conformations, which is not trivial and is computationally demanding. If efficient, low-resource, and reliable methods to generate such conformational ensembles were available, this strategy could become a powerful tool for predicting aggregation propensity. With the rapid advancements in machine learning-based approaches for modeling protein dynamics and conformational ensembles (e.g., AlphaFlow,^59^ ESMFlow,^60^ or BioEmu^61^) we anticipate that our method could become more accessible and practical for routine predictions soon.

## Conclusions

Our study shows the strikingly different effects of the stabilization of two homologous haloalkane dehalogenases on their solubility, aggregation, and unfolding mechanisms By combining in vitro and in silico experiments, we have discovered the mechanisms of their unfolding and revealed the reasons behind the exacerbated aggregation in one of the stabilized proteins, LinB116, compared to the stabilized DhaA115 variant. We revealed the trade-off between stability and solubility caused by the introduced hydrophobic stabilizing mutations. Additionally, we identified the regions in the LinB variants that may be responsible for their aggravated aggregation propensity and the irreversibility of their unfolding. Interestingly, residues hidden in the protein core in the native state may be exposed during unfolding and become important aggregation-prone regions. These residues represent cryptic APRs, which cannot be easily identified in natively folded proteins and require enhanced sampling molecular dynamics simulations. This important observation opens up new possibilities for re-engineering LinB to achieve improved solubility, even at higher protein concentrations, three decades after its discovery.^33^ Our previous attempts to produce this biotechnologically relevant enzyme using fermentation technology were unsuccessful due to protein aggregation at elevated concentrations (unpublished results). Furthermore, for thirty years, we have been intrigued by the striking differences in the solubilities of two highly homologous haloalkane dehalogenases, LinB and DhaA. Our findings are paving the path to novel strategies for engineering LinB, but also other aggregating proteins.^19,51,62,63^ Finally, our study demonstrates the complexity of stability engineering endeavors in the wider scope and the importance of contextual information for protein stabilization by automated platforms.

## Materials & Methods

### Materials and Chemicals

The plasmids encoding the mutant variants were obtained by GeneArt Gene Synthesis (ThermoFisher Scientific, USA). The bacterial strain of *Escherichia coli* BL21­(DE3) prepared in-house was used for the expression of target proteins. The other biological material used in this work includes deoxyribonuclease I—DNase (Thermo Fisher Scientific, USA), which was used during the protein isolation procedure. All chemicals used in this study, classified as of the quality of pro analysis, were purchased from Sigma unless specified otherwise.

### Protein Expression, Purification, and Quality Control

The examined HLDs were expressed in *E. coli* BL21 (DE3) transformed by the plasmids (pET21b) encoding respective proteins. The protein expression was induced by the addition of IPTG (0.4 mM final concentration) and carried out at 20 °C for 16 h. The cells were harvested by centrifugation (4,000*g*, 4 °C, 30 min) and frozen at −70 °C. After defrosting, the resuspended cells were disrupted by the addition of DNase I and 3 rounds of sonication. The disrupted cells were centrifuged (21,000*g*, 4 °C, 60 min), and the proteins were purified from the cell lysates by metallo-affinity chromatography with HisTrap HP column (Cytiva, USA) charged with Ni^2+^ ions using a stepwise increase of imidazole in the elution buffer. The pure monomeric fraction for the following experiments was isolated by size-exclusion chromatography using the Superdex 200 pg 10/300 GL column (Cytiva, USA) with the 50 mM Potassium phosphate buffer (pH 7.5) used as the mobile phase. Protein purity was verified by SDS-PAGE, and the concentration was determined by UV-absorbance measurement (DeNovix DS-11 Spectrophotometer, DeNovix Inc., USA).

### Temperature Scanning Experiments

#### Differential Scanning Fluorimetry (DSF)

The protein unfolding and aggregation were monitored by continual measurement of changes in tryptophan intrinsic fluorescence and static light scattering (SLS), respectively, using the Uncle screening platform (Unchained laboratories, USA). The protein samples (9 μL, concentration ∼ 0.1–1.0 mg/mL) were heated from 20 to 90 °C at different scan rates (0.3 or 0.5, 1, and 2 °C/min). The fluorescence signal, excited at 266 nm, was collected during the experiments. For the estimation of the protein stability, the melting temperature (*T*_m_) was calculated as the average midpoint of 3 independent barycentric mean (BCM) curves plotted against the temperature. In parallel to the protein unfolding, the protein aggregation was estimated from the maximum amplitude of the SLS curve.

#### Differential Scanning Calorimetry (DSC)

The heat capacity changes of the proteins during the unfolding were recorded against the dialysis buffer (50 mM potassium phosphate, 7.5 pH) using MicroCal VP-Capillary DSC (GE Healthcare, USA) from 20 to 90 °C at a 1 °C/min scan rate. The samples were prepared at a concentration of approximately 0.8 mg/mL and thoroughly degassed. The data were corrected by the subtraction of the buffer baseline and normalized to protein concentration prior to further analysis. The melting temperature of each variant was determined as the maximum of the heat capacity peak. Each sample was analyzed in triplicate. The unfolding reversibility was determined from the reheating scan of the sample, which was previously heated up to the temperature where the complete unfolding of the protein was reached (i.e., right after the calorimetric peak) and subsequently cooled down to 20 °C at a 1 °C/min scan rate. The reversibility was then estimated as a ratio of integrated calorimetric peaks of reheated and nonreheated scans.

#### Circular Dichroism (CD)

The secondary structure of the analyzed enzyme variants was verified using circular dichroism (CD) spectroscopy. CD spectra were measured at 20 °C using a spectropolarimeter Chirascan (Applied Photophysics, UK). The samples consisted of 350 μL of the protein (∼0.175 mg/mL) in the 50 mM potassium phosphate buffer (pH 7.5). Data were collected from 190 to 260 nm with 0.25 s integration time and 1 nm bandwidth using a 0.1 cm quartz cuvette. Each spectrum was calculated as an average of five individual repeats after the subtraction of the background. Data were visualized as a mean residue ellipticity (Θ_MRE_) using the following equation

$$\Theta_{MRE}=\frac{\Theta_{OBS}.M_{w}.100}{n.c.l}$$

where Θ_OBS_ stands for the measured ellipticity in degrees, *M*_w_ is the molecular weight of the protein, 100 is the factor originating from the conversion of the molecular weight to mg/dmol, *n* is the number of amino acids, *c* is the protein concentration in mg/mL and *l* is the cell path length in cm. The changes in the secondary structure during temperature-induced unfolding were monitored using CD spectroscopy. The ellipticity at 227 nm was measured while the temperature was gradually increased at different scan rates (0.3 or 0.5, 1, and 2 °C/min) from 20 to 90 °C with 0.25 s integration time and 1 nm bandwidth. The concentration of the samples was approximately. 0.175 mg/mL, and the apparent melting temperature of each variant was assessed from the midpoint of the denaturation curve by fitting to the corresponding unfolding model in the Chirascan software.

#### Unfolding Kinetics

The isothermal unfolding kinetics were measured by monitoring the ellipticity changes at 227 nm at different temperatures from 50 to 70 °C (Chirascan spectropolarimeter, Applied Photophysics, UK). The concentrated sample was diluted ∼10 times by the preheated buffer to reach a final concentration of 0.175 mg/mL and then immediately transferred to a preheated quartz cuvette in the instrument measurement chamber. The whole process took approximately 5–10 s, which accounts for the dead time of the measurement. The precise temperature of the solution was determined by a thermocouple inserted into the cuvette at the end of the measurement. The unfolding kinetics of LinB in the 60–70 °C range were collected using a rapid mixing temperature jump (mT-jump) apparatus mounted on the SFM3000 Stopped-flow chassis (BioLogic, France). Temperature jumps were achieved by mixing the preheated buffer (50 mM potassium phosphate, 7.5 pH) with native LinB at a 4:1 ratio in the total volume of 312 μL, final protein concentration of ∼0.2 mg/mL, and 15 mL/s flow rate. The unfolding kinetics were monitored over the course of 20 s for each jump using changes of LinB tryptophan fluorescence excited at 280 nm and monitored by a PMT tube with a 320 nm cutoff filter, and the final traces are represented as an average of three independent measurements.

#### Global Analysis of Temperature Denaturation Experiments

The selected temperature scanning experiments were analyzed globally using CalFitter 2.0 web server.^41^ The spectroscopy (CD, DSF), calorimetry (DSC), and unfolding kinetics (CD/mT-jump) data were fitted globally to the three-state irreversible model of unfolding. The quality of the fit was sensitive to the dissection of the calorimetric enthalpy between the individual steps, with the best fits being obtained when the Δ*H*_cal_ of one of the two steps was fixed to zero, so all the heat released during the unfolding was assigned to one of the steps. Besides, the effect of Δ*C*p was omitted during the global fitting by using DSC data corrected by the subtraction of a linear baseline connecting the pre- and post-transition signals. This improved the results of fitting but disabled the extrapolation of the values of the kinetic barriers to lower temperatures. The energy barriers (Δ*G* ^⧧^) at specific temperatures (40, 60, and 80 °C) and the calorimetric enthalpies (Δ*H*_cal_) were used for comparison of the thermostability of the variants.

### Molecular Dynamics Simulations

#### System Preparation and Equilibration

The three-dimensional structures of DhaAwt, DhaA115, and LinBwt were obtained from the RCSB Protein Data Bank^64^ (PDB IDs: 4E46, 6SP5, and 1MJ5, respectively). The duplicated side chains of some residues were corrected, leaving the most populated conformation. LinB116 was constructed by homology modeling using the SWISS-MODEL server,^65^ accessible via the Expasy web server. The targeted sequence with the corresponding amino acid substitutions was submitted in FASTA format, and the template search was used to build the model on the crystal structure of the closest variant, LinB32 (PDB ID 4WDQ). The water molecules and ions were removed, and all hydrogen atoms were added using the H++ server,^66^ calculated in an implicit solvent at pH 7.5, 0.1 M salinity, internal dielectric constant of 10, and external of 80. The water molecules from the original crystal structures (or its LinBwt homologue, in the case of LinB116) were added to those structures.

The following steps were performed with the High Throughput Molecular Dynamics (HTMD)^42^ script. The systems were solvated in a cubic water box of TIP3P^67^ water molecules, with the edges at least 15 Å away from the protein by the solvate module of HTMD. Cl^–^ and Na^+^ ions were added to neutralize the charge of the protein and get a final salt concentration of 0.1 M. The system’s topology was built using the charmmbuild module of HTMD, with the modified CHARMM36m^68^ force field and the parameters for the modified mTIP3P^67^ solvent model. The combination CHARMM36m/mTIP3P is expected to provide more accurate ensembles for intrinsically disordered proteins, which is the case when studying the unfolding of proteins.^68^

The systems were equilibrated using the Equilibration_v2 module of HTMD.^42^ The system was first minimized using the conjugate-gradient method for 500 steps. Then the system was heated to 370 K and minimized as follows: (i) 500 steps (2 ps) of NPT thermalization with the Berendsen barostat with 1 kcal·mol^–1^·Å^–2^ constraints on all heavy atoms of the protein, (ii) 1,250,000 steps (5 ns) of NVT equilibration with Langevin thermostat and the same constraints, and (iii) 1,250,000 steps (5 ns) of NVT equilibration with the Langevin thermostat without any constraints. During the equilibration simulations, holonomic constraints were applied on all hydrogen-heavy atom bond terms, and the mass of the hydrogen atoms was scaled by a factor of 4, enabling a 4 fs time step.^69–72^ The simulations employed periodic boundary conditions, using the particle mesh Ewald method for the treatment of interactions beyond 9 Å, and the smoothing and switching of van der Waals interactions was performed for a cutoff of 7.5 Å^71^.

#### Adaptive Sampling

HTMD was used to perform adaptive sampling of the protein unfolding. The 50 ns production MD runs were started with the systems that resulted from the equilibration cycle and employed the same settings as the last step of the equilibration. As suggested by others,^73^ the Langevin collision rate was decreased from 0.1 to 0.02 ps^–1^. The trajectories were saved every 0.1 ns. Adaptive sampling was performed using the RMSD of the C_α_ atoms with respect to the initial structures, excluding the naturally flexible terminal residues (only residues 12–285 were used), and time-based independent component analysis (tICA)^74^ projected in 1 dimension. 40 epochs of 10 MDs each were performed for every system, corresponding to a cumulative simulation time of 20 μs.

#### Markov State Model Construction

The simulations were made into a simulation list using HTMD^42^ the water was filtered out, and any unsuccessful simulations with lengths shorter than 50 ns were omitted. This resulted in 20 μs of cumulative simulation time (400 × 50 ns). The unfolding dynamics were studied by the same metric used in the adaptive sampling: the RMSD of the C_α_ atoms (for residues 12–285). The data was clustered using the MiniBatchKmeans algorithm into 1000 clusters. Thirty or 15 ns lag times were used to construct Markov state models (MSMs) with three or four states (for the DhaA and LinB variants, respectively). The Chapman–Kolmogorov test was performed to assess the quality of the constructed MSMs. Each state was saved as a trajectory XTC file containing 1000 randomly selected representative snapshots; the states were visualized in VMD 1.9.3.^75^ These states were labeled based on their average RMSD values as folded (lowest mean RMSD), intermediate (intermediate RMSD), and unfolded (highest RMSD). The equilibrium population of each state was also obtained from the analysis. For consistency among the different systems, the two intermediate states in the LinB variants (out of the four states obtained) were combined into a single one and used as such in further analysis.

#### Analysis of MDs and States

The cpptraj^76^ module of AmberTools 16 ^77^ was used to concatenate the filtered trajectories of each system by increasing epoch, center, and align them by their backbone atoms, save the combined trajectory in a single file, and compute the time evolution of the RMSD and overall B-factors. Cpptraj was also used to analyze each state from the MSMs and compute several parameters: the respective RMSD of and *B*-factors of C_α_ or backbone atoms, and the solvent-accessible surface area (SASA) of the protein, using the LCPO^78^ algorithm. Unless stated, these calculations were performed for residues 12–285 (without the flexible terminal regions). The relative accessible surface area (rASA) was calculated for the same states as rASA = SASA/SASA^Max^, where SASA^Max^ is the maximum possible solvent accessible surface area for the individual amino acid residues, as obtained from Tien et al.^79^ We considered a residue to be exposed for rASA ≥0.25.^80^

#### Aggregation Propensity Predictions

Prediction of aggregation-prone regions (APRs) was performed with the AggreProt Web server^50^ for the LinB and DhaA variants. The sequences of the analyzed HLDs were used as the input, together with the corresponding structure files. In the case of DhaAwt, DhaA115, and LinBwt, the experimental structures were used (PDB IDs: 4HZG, 6SP5, and 1MJ5), while the structure of LinB116 was represented by the structure model predicted using homology modeling by SWISS-MODEL^65^ (as described above). The aggregation profiles were subsequently exported and analyzed in the context of other experiments.

The Aggrescan3D 2.0 ^51^ server was used to evaluate the aggregation propensity of LinB116. Ten random snapshots (out of the 1000 in total) of the most extensively unfolded state were submitted. For each snapshot, two separate analyses were conducted. One employed the Static mode, and the other the Dynamic mode. The additional parameters that were selected while using the Static mode are as follows: Stability calculations—yes, Dynamic mode—no, Distance of aggregation −5 Å, FoldX usage—yes, Automated mutations—yes. In the case of the Dynamic mode, the parameters were set as follows: Stability calculations—yes, Dynamic mode—yes, Distance of aggregation −5 Å, FoldX usage—yes, Automated mutations—no. The same parameters and exclusion criteria were used in the analysis of both the wild-type and the variant.

## Supplementary Material



## Notes

Figure S1: Spectra of circular dichroism of tested HLDs; Figure S2: Concentration dependence of the stability of the tested proteins; Figure S3: Evolution of RMSD in time during the unfolding adaptive sampling simulations; Figure S4: Implied time scale plots for the MSM analysis of the studied proteins; Figure S5: Chapman–Kolmogorov tests for the MSMs of the studied proteins; Figure S6: Structural ensembles of the states in the studied proteins; Figure S7: Properties of the different states in the studied proteins; Figure S8: Global residue flexibility assessed by the total RMSD during the adaptive simulations; Figure S9: State flexibility assessed by the *B*-factors; Figure S10: *B*-factors of the intermediate states; Figure S11: Main changes in the solvent exposure; Figure S12: The aggregation propensity profiles; Figure S13: Structural context of aggregation hot-spots in LinB116; Figure S14: Experimental overview of tested mutational variants of LinB116; Table S1: Overview of the expressibility of the tested HLDs and their specific activities; Table S2: The overview of the melting temperatures of tested protein in various concentrations; Table S3: Properties of the different states in the studied proteins; Table S4: Experimental and computational secondary structures; Table S5: The overview of the hydrophobicity changes upon the stabilization; Table S6: Overview of aggregation hot spots, identified using AggreScan3D 2.0; Table S7: Final designs of LinB116; Table S8: Overview of the experimental characterization of LinB116 variants (PDF)

## ∥

Protein Biophysics, Department of Biotechnology and Biomedicine, Technical University of Denmark, Søltofts Plads, Building 227, 2800 Kgs. Lyngby, Denmark
