# Supplementary material for: Decoding Protein Stabilization: Impact on Aggregation, Solubility, and Unfolding Mechanisms
Source: J Chem Inf Model. 2025 Aug 6;65(16):8688–701. doi: 10.1021/acs.jcim.5c00611 (PMC12381856; doi:10.1021/acs.jcim.5c00611)
Supplement: Supplementary file 1 [file ci5c00611_si_001.pdf]

Probing Protein Stabilization Effects on Aggregation, Solubility, and Unfolding Mechanism

Martin Havlásek,[1,2] Sérgio M. Marques,[1,2] Veronika Szotkowská,[1] Antonín Kunka,[1,2, §] Petra Babková,[1,2] Jiří Damborský,[1,2] Zbyněk Prokop,[1,2,*] David Bednář[1,2,*]

Affiliations:
[1] Loschmidt Laboratories, Department of Experimental Biology and RECETOX, Faculty of Science, Masaryk University, Kotlarska 2, Brno, Czech Republic

[2] International Clinical Research Centre, St. Anne's University Hospital, Pekarska 53, Brno, Czech Republic

[3] The current affiliation: Protein Biophysics, Department of Biotechnology and Biomedicine, Technical University of Denmark, Søltofts Plads, Building 227, 2800 Kgs. Lyngby, Denmark

* Corresponding authors: ZP - zbynek@chemi.muni.cz, DB – davidbednar1208@gmail.com

Supplementary material

Table of Figures

Figure S1: Spectra of circular dichroism of tested HLDs.. 2
Figure S2: Concentration dependence of the stability of the tested proteins. 2
Figure S3: Evolution of RMSD in time during the unfolding adaptive sampling simulations.. 3
Figure S4: Implied timescale plots for the MSM analysis of the studied proteins.. 3
Figure S5: Chapman-Kolmogorov tests for the MSMs of the studied proteins. 4
Figure S6: Structural ensembles of the states in the studied proteins. 5
Figure S7: Properties of the different states in the studied proteins. 6
Figure S8: Global residue flexibility assessed by the total RMSD during the adaptive simulations 6
Figure S9: State flexibility assessed by the B-factors 7
Figure S10: B-factors of the intermediate states. 8
Figure S11: Main changes in the solvent exposure 9
Figure S12: The aggregation propensity profiles 10
Figure S13: Structural context of aggregation hot-spots in LinB116 11
Figure S14: Experimental overview of tested mutational variants of LinB116. 12

Table of tables

Table S1: Overview of the expressibility of the tested HLDs and their specific activities. 13
Table S2: The overview of the melting temperatures of tested protein in various concentrations. 13
Table S3: Properties of the different states in the studied proteins. 13
Table S4: Experimental and computational secondary structures (SS) 14
Table S5: The overview of the hydrophobicity changes upon the stabilization 15
Table S6: Overview of aggregation hot spots, identified using AggreScan3D 2.0. 16
Table S7: Final designs of LinB116. 17
Table S8: Overview of the experimental characterization of LinB116 variants. 18

Supplementary Figures

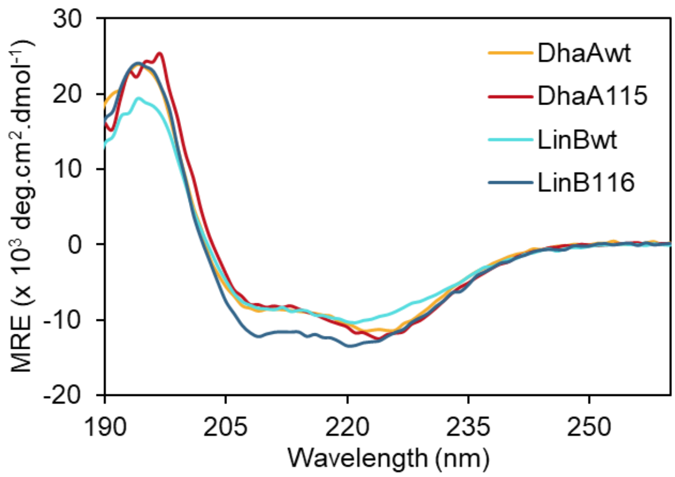

Figure S1: Spectra of circular dichroism of tested HLDs. All measured proteins displayed one positive peak at 195−197 nm and two negative minima at approximately 209 and 224 nm, which are characteristics typical for an α/β-hydrolase fold suggesting their proper folding and high structural similarity.

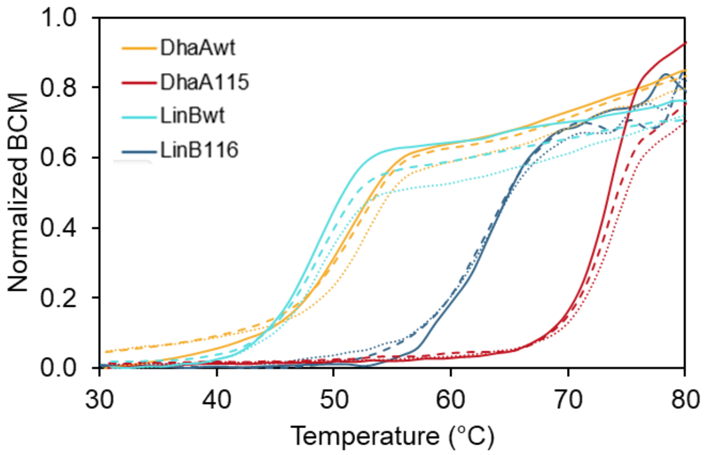

Figure S2: Concentration dependence of the stability of the tested proteins measured using DSF. Dotted lines represent traces of samples with a concentration of 0.1 mg/mL, dashed lines of 0.5 mg/mL, and full lines of 1.0 mg/mL.

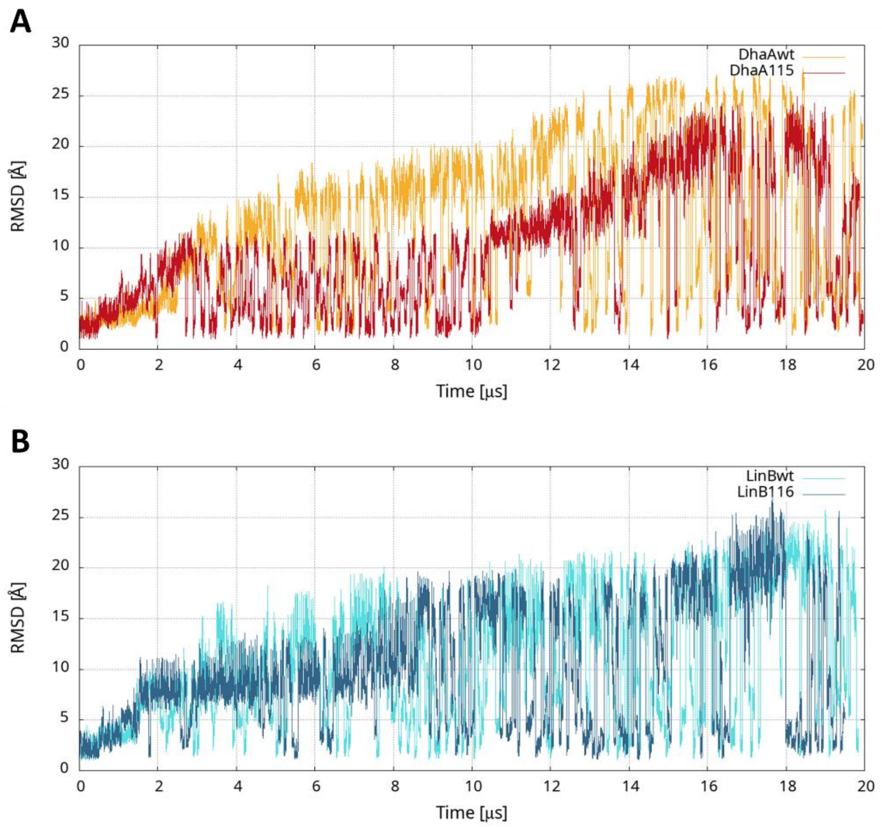

Figure S3: Evolution of RMSD in time during the unfolding adaptive sampling simulations with the studied proteins. A) DhaAwt and DhaA115, and B) LinBwt and LinB116. The RMSD values were calculated for the C_α atoms of the protein in comparison with the respective initial structures.

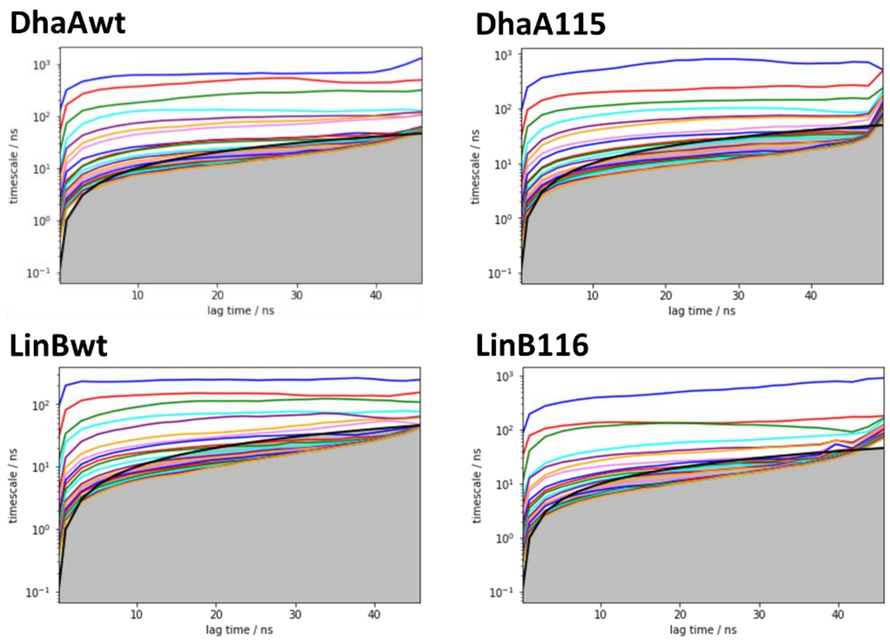

Figure S4: Implied timescale plots for the MSM analysis of the studied proteins. Plots for DhaAwt, DhaA115, LinBwt and LinB116. The plots show the convergence of the timescales of the slowest transitions in the MSM for lag times higher than the selected values of 30 ns (DhaAwt and DhaA115) or 15 ns (LinBwt and LinB116).

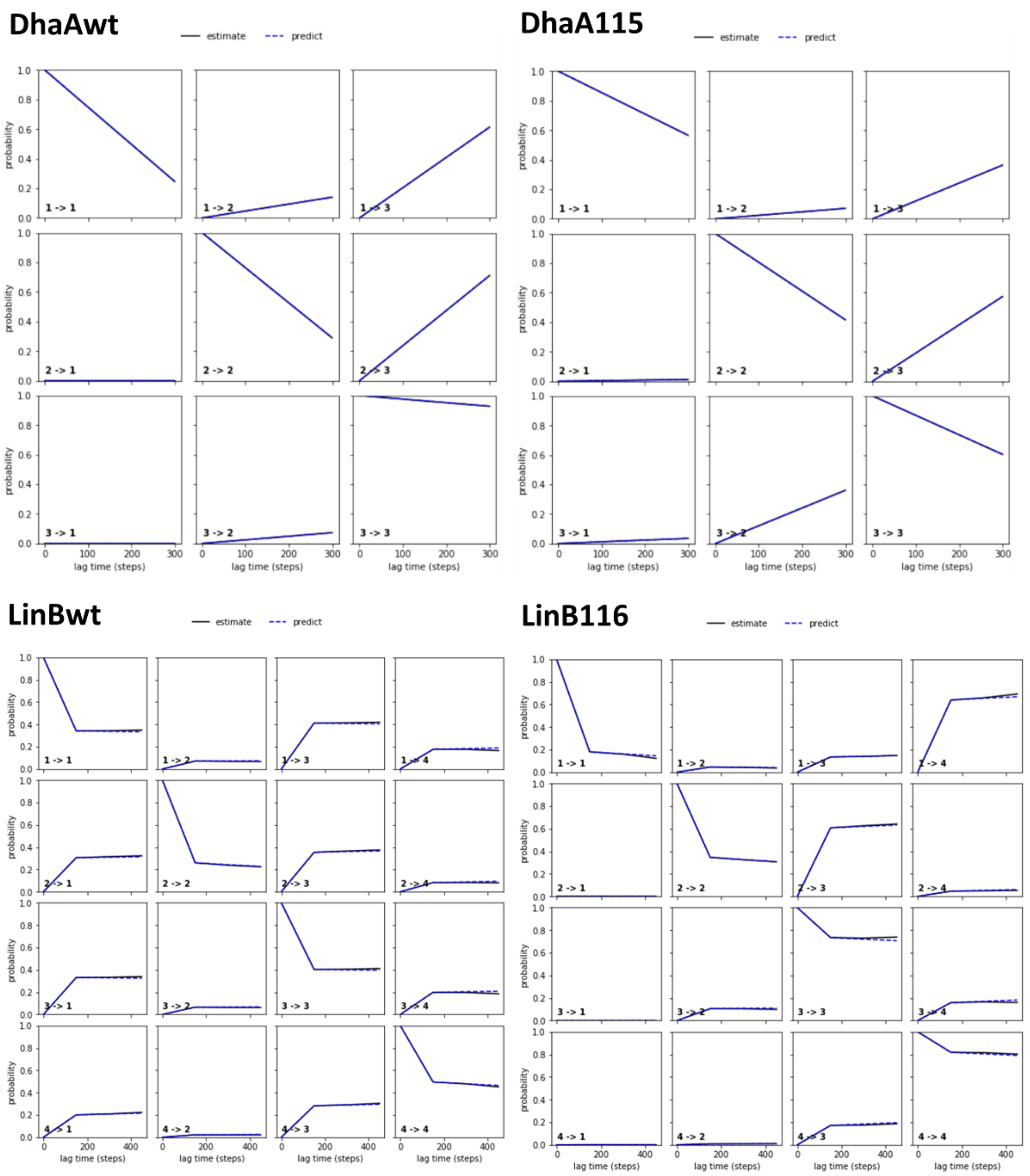

Figure S5: Chapman-Kolmogorov tests for the MSMs of the studied proteins. Transition probability plots for the 3-state MSMs of DhaAwt and DhaA115, or the 4-state MSMs of LinBwt and LinB116. The good superimposition of the "estimate" with the "predict" transition probability curves show that the models are Markovian for the respective selected lag times.

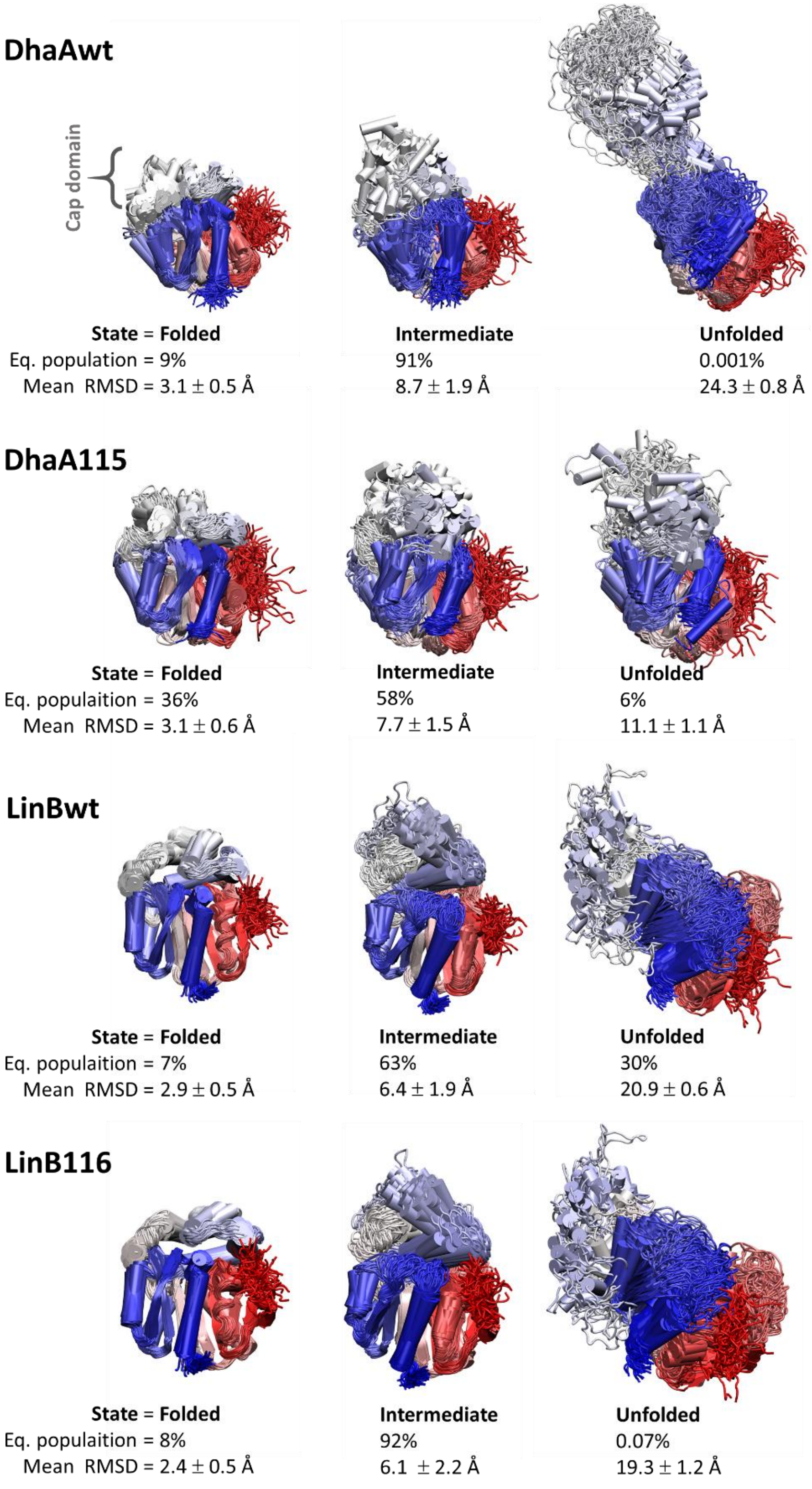

Figure S6: Structural ensembles of the states in the studied proteins. Clusters obtained from the MSMS analysis for DhaAwt, DhaA115, LinBwt and LinB116, describing the folded, intermediate and unfolded states. The protein is represented in cartoons, the red-white-blue color gradient shows the residue sequence index (red = N-, blue = C-terminus), and the cap domain (white region) is labelled. Each state is represented by 100 randomly selected frames superimposed and aligned to the crystal structures. The predicted equilibrium population and mean RMSD of the C_α atoms is reported for each state.

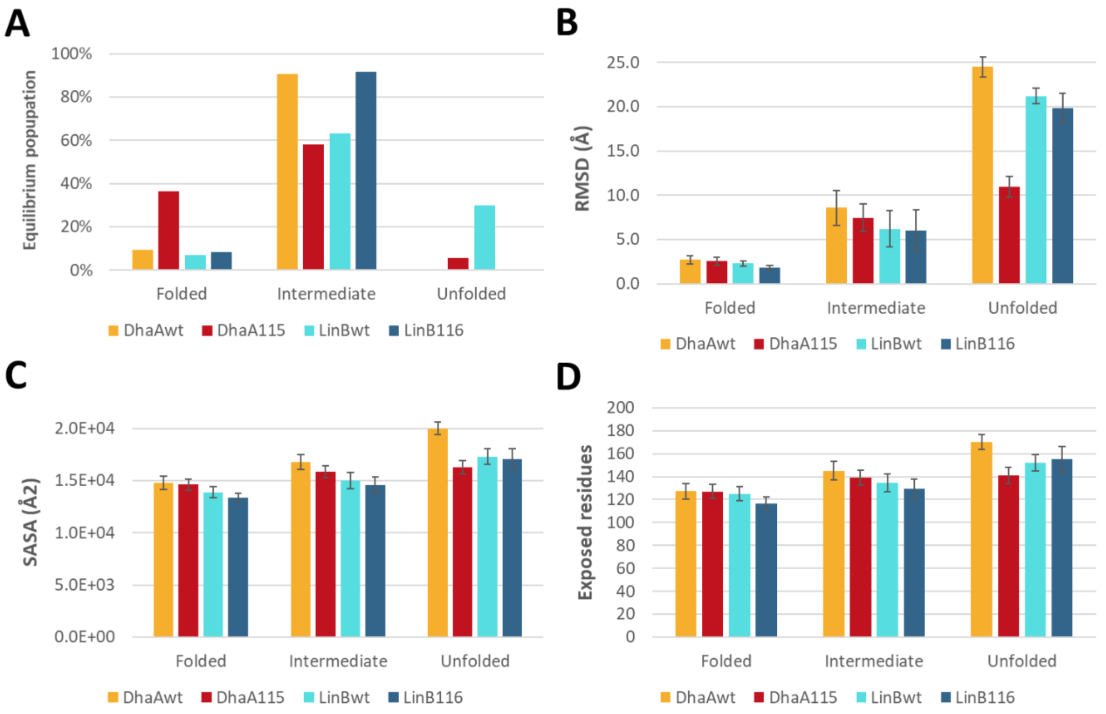

Figure S7: Properties of the different states in the studied proteins. A) Estimated equilibrium population, B) mean RMSD values of the Cα atoms with respect to the respective crystal structures, C) mean SASA, and D) number of exposed residues (with rASA ≥ 0.25). The error bars represent the standard deviation over each ensemble (consisting of 1000 frames for most states, and 2000 frames for the intermediate states of the LinB variants).

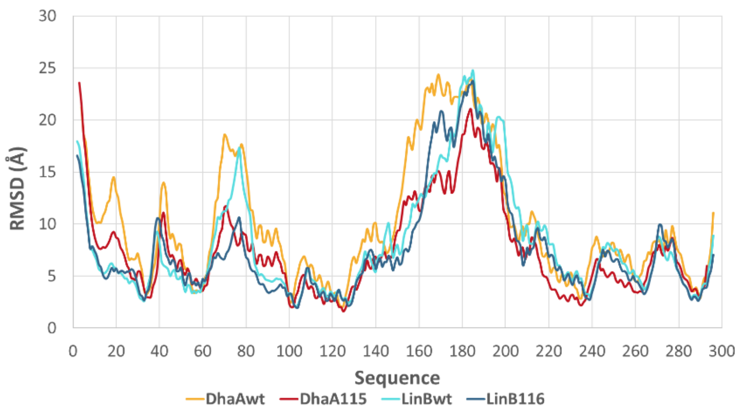

Figure S8: Global residue flexibility assessed by the total RMSD during the adaptive simulations with the studied proteins. RMSD by residue computed for the backbone atoms (C, C_α, N, O) of the respective residues during the total extension of the MD simulations performed for each protein.

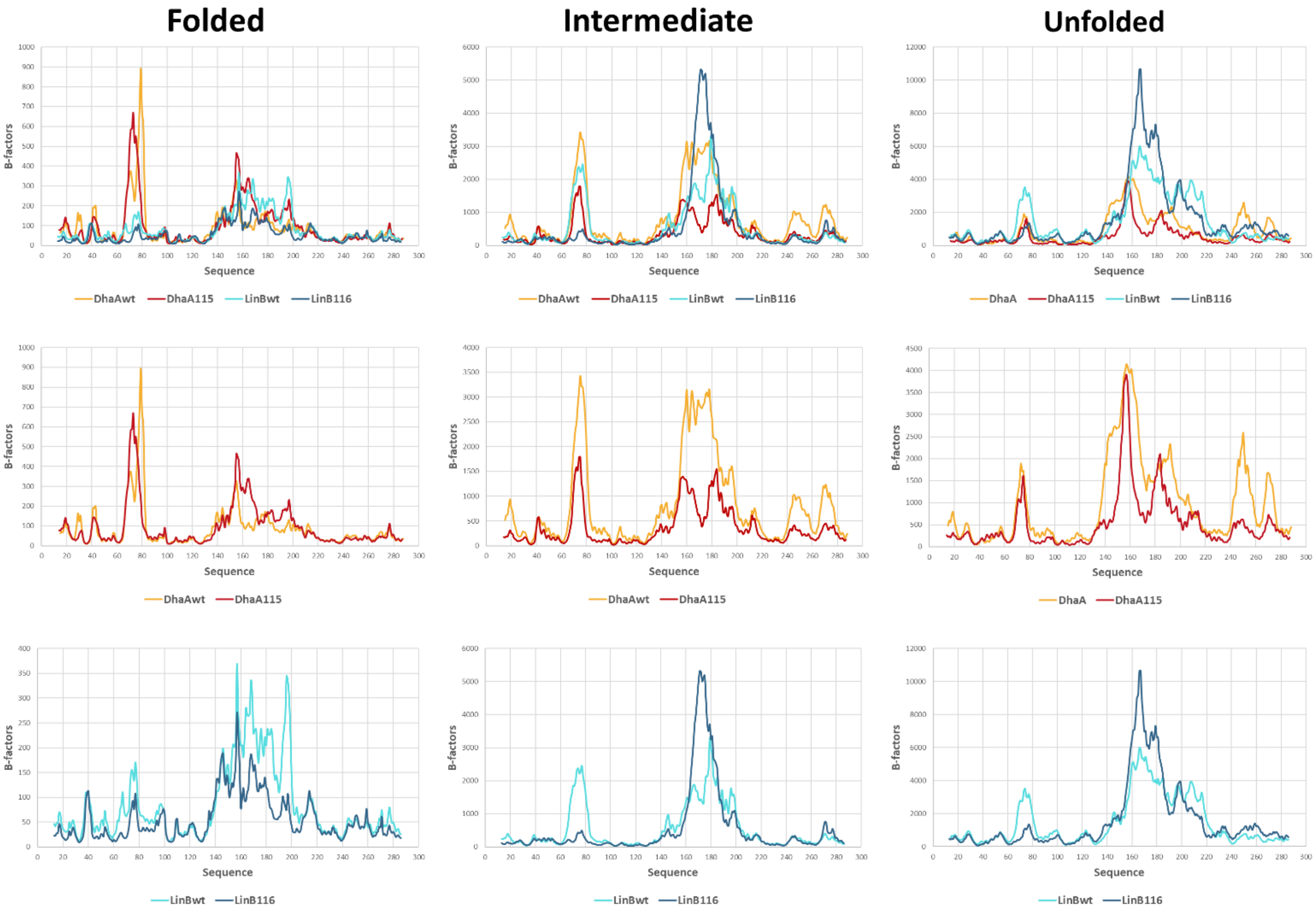

Figure S9: State flexibility assessed by the B-factors within each state of the studied proteins. B-factors of the backbone atoms (C, Cα, N, O) of each residue, for the folded, intermediate and unfolded states, superimposing all the proteins (top), only the DhaA variants (central row), and only the LinB variants (bottom).

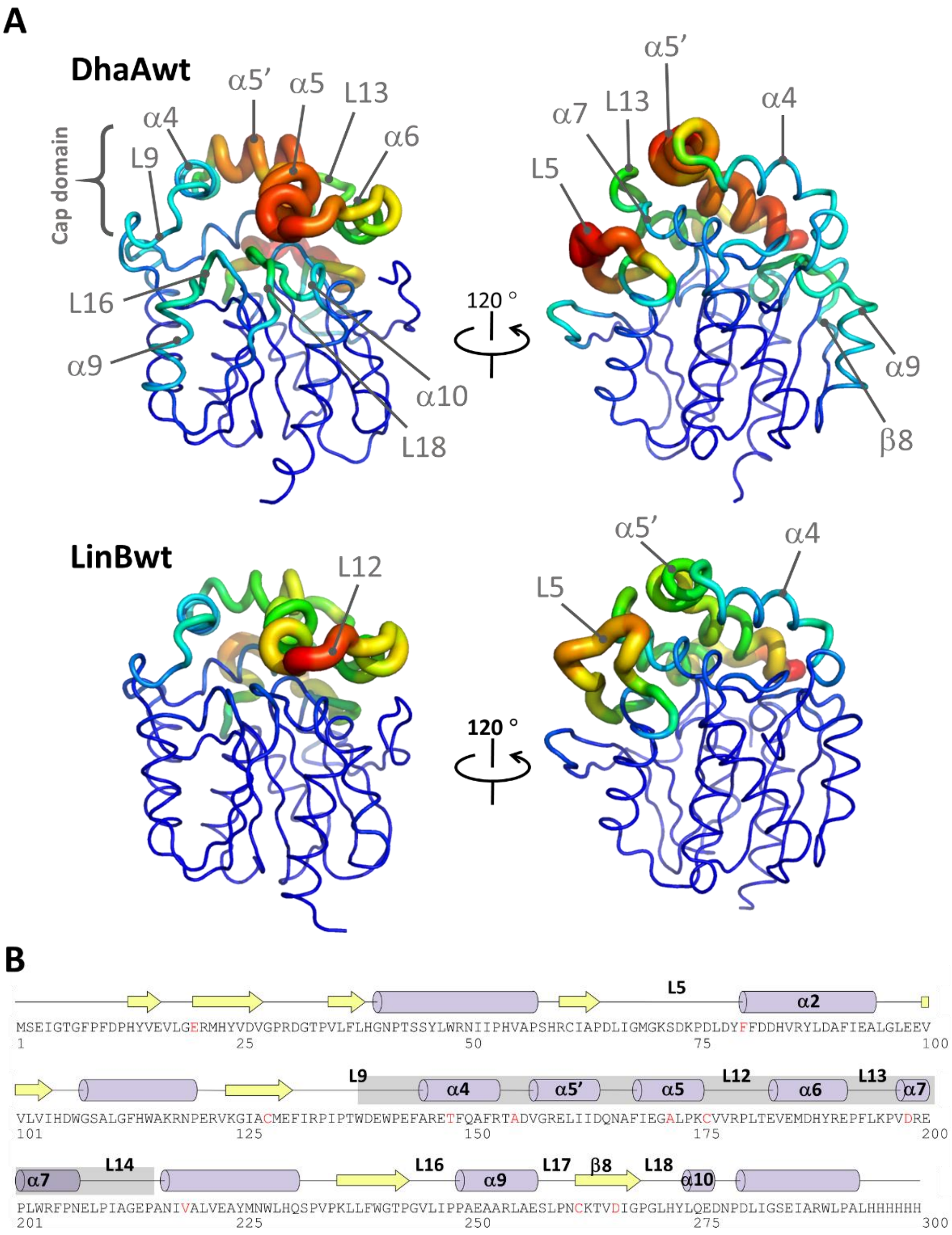

Figure S10: B-factors of the intermediate states of DhaAwt and LinBwt and secondary structure representation. A) Structural representation of the B-factors of the backbone atoms (C, C_α, N, O) in the intermediate states for DhaAwt and LinBwt. The structures correspond to the respective crystal structures (PDB ID 4E46 for DhaAwt, and PDB ID 1MJ5 for LinBwt); the hottest colors (red) and thickest tubes correspond to the highest B-factor values in each system, and the blue and thinnest to the lowest values, and the most flexible regions are labeled. B) Secondary structure elements represented on the sequence of DhaAwt, including β-strands (yellow arrows), α-helices (purple barrels), and loops (black lines), where the most flexible regions indicated above are labeled. The cap domain is highlighted by the grey box, and the positions where mutations were introduced to generate DhaA115 are in red.

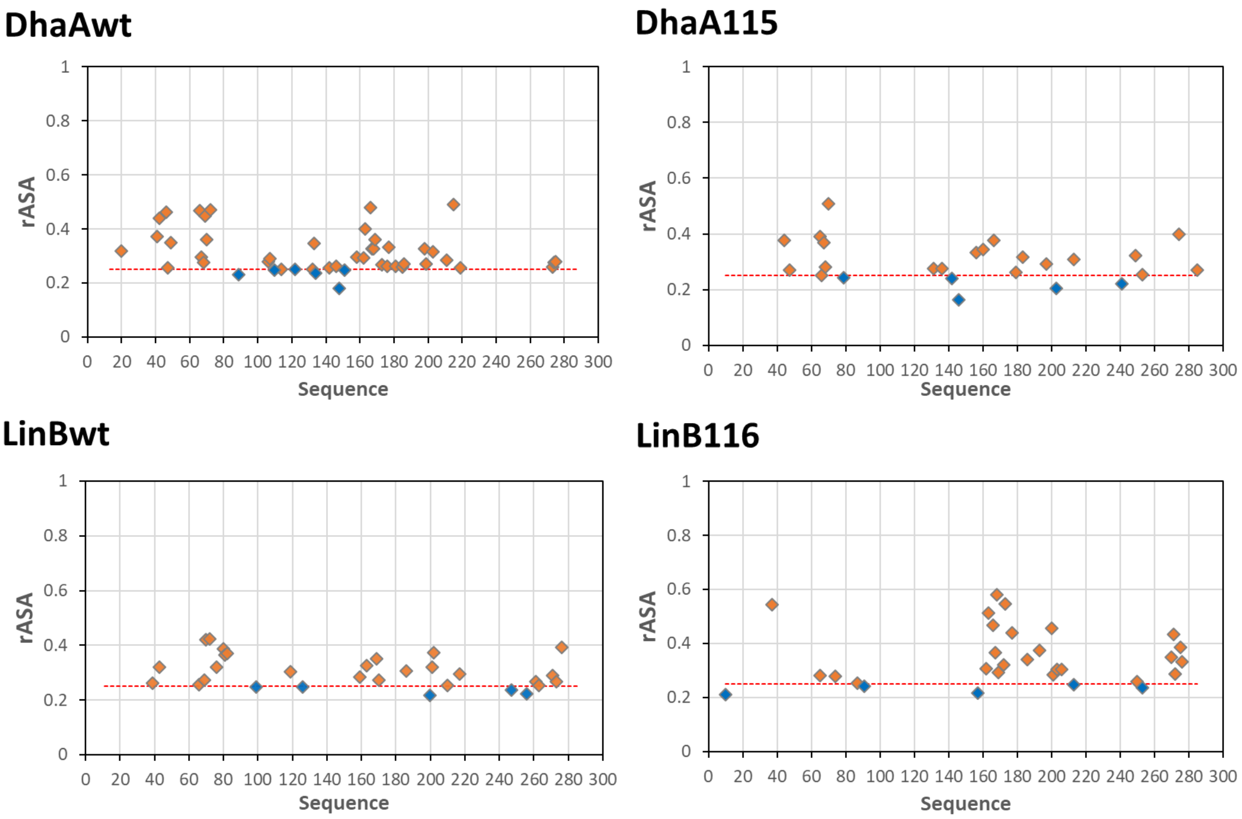

Figure S11: Main changes in the solvent exposure from the folded to the intermediate states of the studied proteins. RASA of the residues that change their state from buried to exposed (orange diamonds) and those that changed from exposed to buried (blue diamonds). The threshold for the solvent-exposure classification (rASA = 0.25) is represented by the red dotted line.

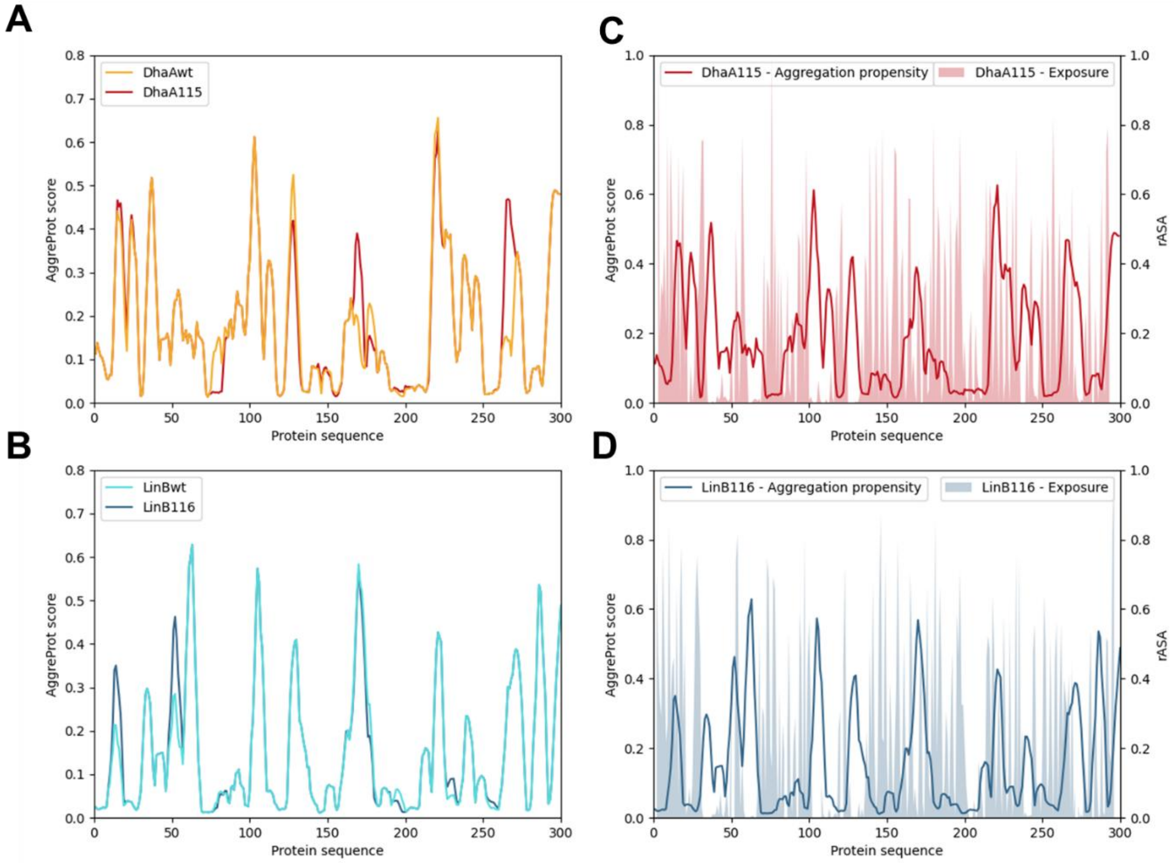

Figure S12: The aggregation propensity profiles of DhaAwt and DhaA115 (A) and LinBwt and LinB116 (B) predicted using AggreProt. In both cases, the profiles of the stabilized variants displayed slightly higher aggregation propensity in some regions. The aggregation propensity of stabilized variants DhaA115 **(C)** and LinB116 **(D)** visualized in the context of solvent accessibility (rASA), as calculated by AggreProt for the respective structures.

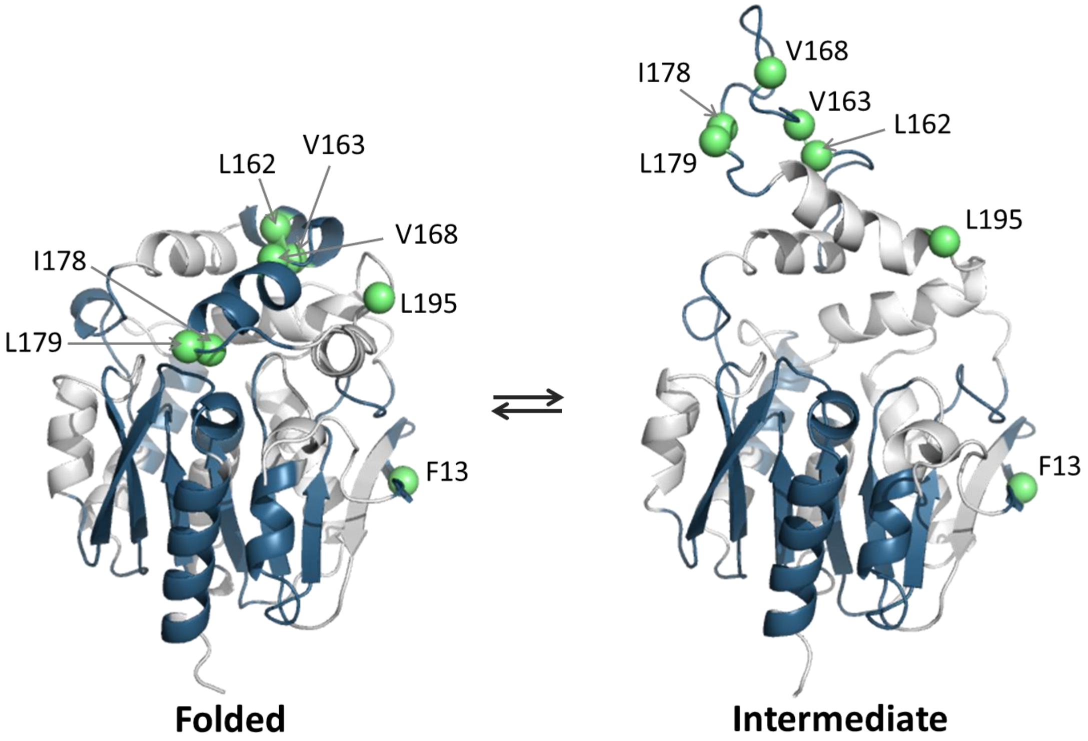

Figure S13: Structural context of aggregation hot-spots in LinB116 identified using AggreScan3D 2.0[1] (A3D, green spheres) and compared with APRs predicted using AggreProt[2] (dark blue).

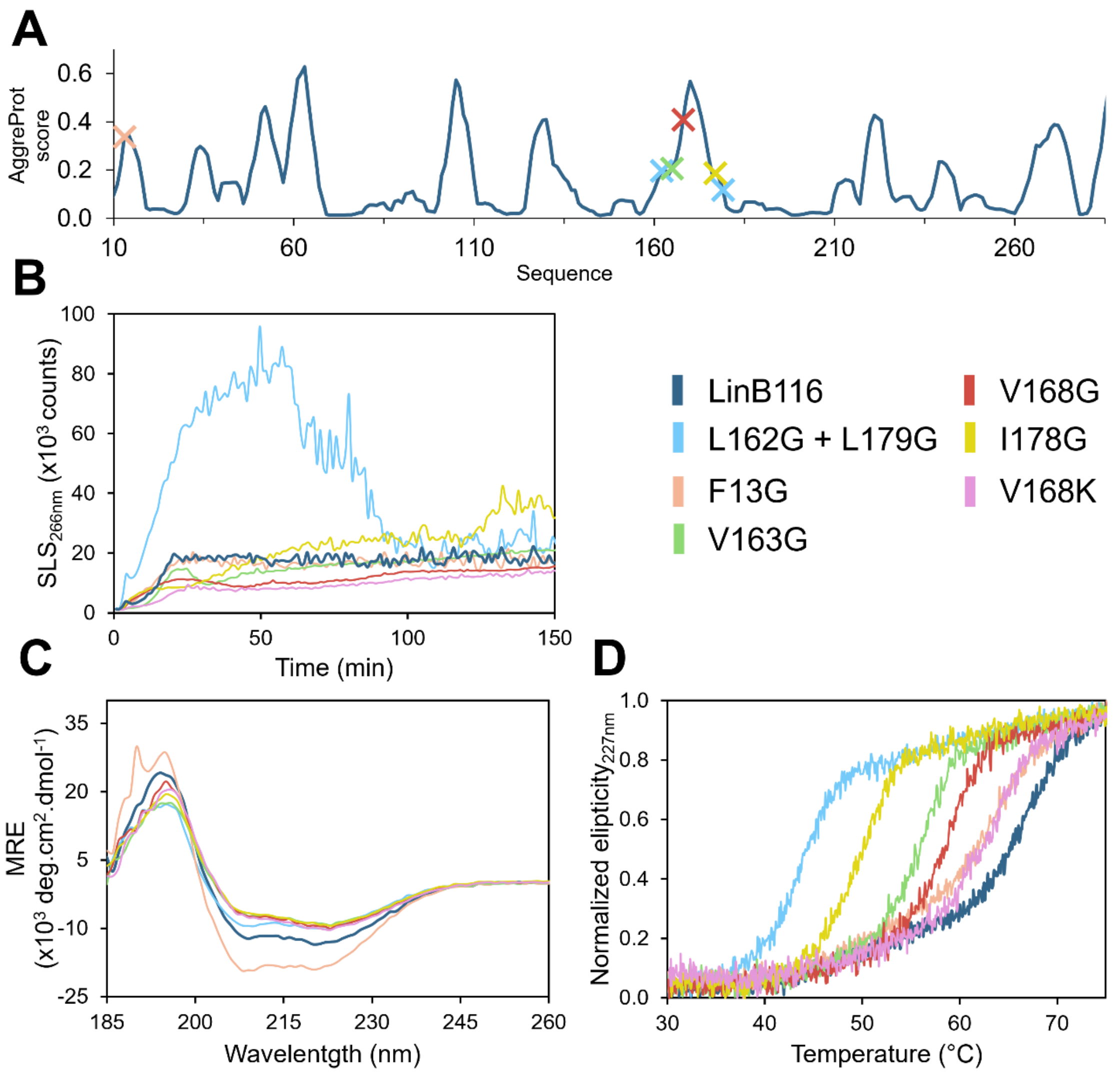

Figure S14: Experimental overview of tested mutational variants of LinB116. (A) Aggregation profile of LinB116 predicted using AggreProt. The corresponding mutations are highlighted by color-coded crosses**. (B)** SLS aggregation kinetics showed no variant with suppressed aggregation. **(B)** CD spectra confirmed secondary structure content typical for haloalkane dehalogenases. **(C)** Unfolding curves from CD displayed a destabilizing effect of all designed mutations.

Supplementary Tables

Table S1: Overview of the expressibility of the tested HLDs and their specific activities towards 1,2-dibromoethane and 1-iodohexane. Activity values of DhaA variants with 1-iodohexane are taken from the previous study[3]. The yields were estimated as the average of the yields from at least 2 independent batches with errors corresponding to standard deviations from the individual measurements.

Variant	Yield (mg/mL)	Specific activity ($nmol.mg^{-1}.s^{-1}$)	
		1,2-dibromoethane	1-iodohexane
DhaAwt	85.0 ± 8.5	64.8	18.0
DhaA115	66.0 ± 29.7	7.6	5.6
LinBwt	13.5 ± 2.1	133.4	46.0
LinB116	6.5 ± 2.1	16.6	16.1

Table S2: The overview of the melting temperatures of tested protein in various concentrations. The results show no significant changes in the stability with changed concentration. The uncertainty represents the standard deviation.

Variant	T_m^{app} (°C)		
	0.1 (mg/mL)	0.5 (mg/mL)	1.0 (mg/mL)
DhaAwt	52.8 ± 0.2	51.7 ± 0.3	51.1 ± 0.1
DhaA115	74.9 ± 1.4	73.5 ± 0.2	73.6 ± 0.1
LinBwt	48.9 ± 0.1	48.9 ± 0.1	48.6 ± 0.0
LinB116	63.4 ± 0.0	63.2 ± 0.1	63.1 ± 0.7

Table S3: Properties of the different states in the studied proteins. Estimated equilibrium population, mean RMSD of the Cα atoms with respect to the respective crystal structures, mean SASA, and number of exposed residues (with rASA ≥ 0.25).[a]

State	Enzyme	State population	RMSD (Å)	SASA ($Å^2$)	No. exposed residues
Folded	DhaAwt	9%	2.7 ± 0.5	14788 ± 614	127.2 ± 6.9
	DhaA115	36%	2.5 ± 0.5	14648 ± 537	127.0 ± 6.2
	LinBwt	7%	2.3 ± 0.3	13892 ± 543	125.0 ± 6.1
	LinB116	8%	1.8 ± 0.3	13384 ± 439	116.6 ± 5.3
Intermediate	DhaAwt	91%	8.6 ± 2.0	16802 ± 706	145.1 ± 7.8
	DhaA115	58%	7.4 ± 1.6	15856 ± 592	138.8 ± 6.5
	LinBwt	63%	6.2 ± 2.1	15015 ± 798	134.6 ± 7.9
	LinB116	92%	6.0 ± 2.3	14604 ± 749	129.4 ± 8.6
Unfolded	DhaAwt	0.001%	24.5 ± 1.1	20024 ± 619	170.2 ± 6.3
	DhaA115	6%	11.0 ± 1.2	16294 ± 636	141.0 ± 7.1
	LinBwt	30%	21.2 ± 0.9	17320 ± 725	151.9 ± 7.2
	LinB116	0.07%	19.8 ± 1.7	17077 ± 957	155.0 ± 11.0

[a]Values calculated for residues 12-285. The uncertainty represents the standard deviation over each ensemble (consisting of 1000 frames for most states, and 2000 frames for the intermediate states of the LinB variants).

Table S4: Experimental and computational secondary structures (SS) of the folded and unfolded DhaA variants. SS content (in %) were obtained experimentally from CD measurements analyzed using BeStSel[4], and computationally from the MSM analysis of our MD simulations, for the native, intermediate (not measurable experimentally), and unfolded states.

Experimental				
	**DhaAwt**		**DhaA115**	
	**Native**	**Unfolded**	**Native**	**Unfolded**
Helix	38	17	34	19
Strand	11	14	14	21
Turn	9	11	9	13
Others*	42	58	43	47

Computational						
	**DhaAwt**			**DhaA115**		
	**Native**	**Intermediate**	**Unfolded**	**Native**	**Intermediate**	**Unfolded**
Helix	42	36	28	41	38	36
Strand	18	16	15	19	18	17
Coil	40	47	57	40	44	47

*The term "others" includes 3,10-helices, π-helices, β-bridges, bends, loops/irregular and invisible regions of the structure.

Table S5: The overview of the hydrophobicity changes of the mutations introduced into DhaA115 and LinB116. The positions on the surface (rASA ≥ 0.25) are highlighted in green and the mutations increasing the hydrophobicity are marked in red.* The total sum of the surface hydrophobicity changes and the relative increase in the total surface hydrophobicity upon the mutations on the surface is positive and higher for LinB116, suggesting a more pronounced decrease in the general solubility after the mutations for this variant.

DhaA115					
**Mutation**			**rASA**	**K.D.**	**Monera**
E	20	S	0.23		
F	80	R	0.30	-7.30	-114
C	128	F	0.01		
**T**	**148**	**L**	**0.27**	**4.50**	**84**
A	155	P	0.66	-3.4	-87
A	172	I	0.07		
C	176	F	0.09		
D	198	W	0.17		
V	219	W	0.11		
C	262	L	0.17		
**D**	**266**	**F**	**0.49**	**6.30**	**155**
**Sum of total surface hydrophobicity change**				**0.1**	**38**
**Relative total surface hydrophobicity change**				**0.1%**	**4%**

LinB116					
**Mutation**			**rASA**	**K.D.**	**Monera**
**E**	**15**	**T**	**0.61**	**2.80**	**44**
**A**	**53**	**L**	**0.63**	**2.00**	**56**
A	81	K	0.23		
**D**	**166**	**K**	**0.56**	**-0.40**	**32**
L	177	W	0.08		
**E**	**192**	**K**	**0.88**	**-0.40**	**8**
A	197	P	0.53	-3.40	-87
G	229	Q	0.42	-3.10	-10
**D**	**255**	**A**	**0.59**	**5.30**	**96**
**Sum of total surface hydrophobicity change**				**2.8**	**139**
**Relative total surface hydrophobicity change**				**1.5%**	**21%**

*Two different hydrophobicity scales were used: K.D. stands for Kyte and Doolittle scale[5] and Monera stands for the scale published by Monera et al.[6].

Table S6: Overview of aggregation hot spots, identified using AggreScan3D 2.0[1] (A3D) from the structures of the unfolding intermediate of LinB116.

Residue	A3D score av	Mutation	ΔΔG (kcal.mol^{-1})
F13	1.8	F13P	6.4
		F13G	4.3
L162	1.6	L162P	12.2
		L162G	5.9
V163	2.2	V163P	17.6
		V163G	7.6
V168	1.8	V168P	14.3
		V168G	4.9
I178	2.2	I178P	22.6
		I178G	7
L179	1.7	L179P	6
		L179G	2.7
L195	1.7	L195P	7
		L195G	2.5

* "A3D score av" refers to the average of A3D predictions from 10 random partially unfolded structures of LinB116 obtained from molecular dynamics simulations. Stability changes caused by the potential mutations at these positions were predicted using Rosetta ddg_monomer[7], following a protocol that was previously described[8].

Table S7: Overview of mutations and designs targeting cryptic APRs of LinB116.

Variant	Mutations	Reason for selection
LinB150	V163G + V168G + I178G	All residues getting exposed during the first phase of unfolding mutated to the least destabilizing glycines
LinB151	F13G + L162G + L179G + L195G	All residues not getting exposed during the first phase of unfolding mutated to the least destabilizing glycines
LinB152	L162G + L179G	Subset of mutations in LinB151 (residues in the next mutant are at very similar positions, but do get exposed)
LinB153	V163G + I178G	A subset of mutations in LinB150
LinB154	V163E + V168K + I178D	Residues getting exposed during the first phase of unfolding, mutated to charged AA
LinB155	L162E + V168P + I178G	Valine at position 168 mutated to proline (residue is positioned at the end of a helix)
LinB156	F13G	Residue in the main domain, single-point mutation to minimize destabilization and observe effect on aggregation
LinB157	V163G	Exposed during unfolding, single-point mutation to minimize destabilization and observe effect on aggregation
LinB158	V168G	Exposed during unfolding, single-point mutation to minimize destabilization and observe effect on aggregation
LinB159	I178G	Exposed during unfolding, single-point mutation to minimize destabilization and observe effect on aggregation
LinB160	V168K	Exposed during unfolding, single-point mutation to minimize destabilization and observe effect on aggregation.

* All variants carry all mutations introduced previously into LinB116.

Table S8: Overview of the experimental characterization of LinB116 variants.

Variant	Yield (mg/L)	T_m^{app} (°C)			ΔT_m^{app} (°C)
LinB116	5	66.5	±	0.1	0
LinB150	No soluble expression				
LinB151	No soluble expression				
LinB152	6	43.7	±	0.0	-22.8
LinB153	No soluble expression				
LinB154	No soluble expression				
LinB155	No soluble expression				
LinB156	3	62.9	±	0.1	-3.6
LinB157	6	55.7	±	0.1	-10.8
LinB158	6	58.3	±	0.1	-8.2
LinB159	6	49.8	±	0.1	-16.7
LinB160	6	62.7	±	0.1	-3.8

References

(1) Kuriata, A.; Iglesias, V.; Pujols, J.; Kurcinski, M.; Kmiecik, S.; Ventura, S. Aggrescan3D (A3D) 2.0: Prediction and Engineering of Protein Solubility. *Nucleic Acids Res.* **2019**, *47* (W1), W300–W307. https://doi.org/10.1093/nar/gkz321.

(2) Planas-Iglesias, J.; Borko, S.; Swiatkowski, J.; Elias, M.; Havlasek, M.; Salamon, O.; Grakova, E.; Kunka, A.; Martinovic, T.; Damborsky, J.; Martinovic, J.; Bednar, D. AggreProt: A Web Server for Predicting and Engineering Aggregation Prone Regions in Proteins. *Nucleic Acids Res.* **2024**, *52* (W1), W159–W169. https://doi.org/10.1093/nar/gkae420.

(3) Bednar, D.; Beerens, K.; Sebestova, E.; Bendl, J.; Khare, S.; Chaloupkova, R.; Prokop, Z.; Brezovsky, J.; Baker, D.; Damborsky, J. FireProt: Energy- and Evolution-Based Computational Design of Thermostable Multiple-Point Mutants. *PLoS Comput. Biol.* **2015**, *11* (11), e1004556. https://doi.org/10.1371/journal.pcbi.1004556.

(4) Micsonai, A.; Moussong, É.; Wien, F.; Boros, E.; Vadászi, H.; Murvai, N.; Lee, Y.-H.; Molnár, T.; Réfrégiers, M.; Goto, Y.; Tantos, Á.; Kardos, J. BeStSel: Webserver for Secondary Structure and Fold Prediction for Protein CD Spectroscopy. *Nucleic Acids Res.* **2022**, *50* (W1), W90–W98. https://doi.org/10.1093/nar/gkac345.

(5) Kyte, J.; Doolittle, R. F. A Simple Method for Displaying the Hydropathic Character of a Protein. *J. Mol. Biol.* **1982**, *157* (1), 105–132. https://doi.org/10.1016/0022-2836(82)90515-0.

(6) Monera, O. D.; Sereda, T. J.; Zhou, N. E.; Kay, C. M.; Hodges, R. S. Relationship of Sidechain Hydrophobicity and Alpha-Helical Propensity on the Stability of the Single-Stranded Amphipathic Alpha-Helix. *J. Pept. Sci. Off. Publ. Eur. Pept. Soc.* **1995**, *1* (5), 319–329. https://doi.org/10.1002/psc.310010507.

(7) Kellogg, E. H.; Leaver-Fay, A.; Baker, D. Role of Conformational Sampling in Computing Mutation-Induced Changes in Protein Structure and Stability. *Proteins* **2011**, *79* (3), 830–838. https://doi.org/10.1002/prot.22921.

(8) Kunka, A.; Marques, S. M.; Havlasek, M.; Vasina, M.; Velatova, N.; Cengelova, L.; Kovar, D.; Damborsky, J.; Marek, M.; Bednar, D.; Prokop, Z. Advancing Enzyme's Stability and Catalytic

Efficiency through Synergy of Force-Field Calculations, Evolutionary Analysis, and Machine Learning. *ACS Catal.* **2023**, *13* (19), 12506–12518. https://doi.org/10.1021/acscatal.3c02575.
